# Isothermal confined pyrolysis on source rock and kerogens in the presence and absence of water: Implication in isotopic rollover in shale gases

**DOI:** 10.1038/s41598-020-62790-6

**Published:** 2020-03-31

**Authors:** Hao Xu, Changchun Pan, Lifei Zeng, Wenkui Huang, Chenxi Zhou, Shuang Yu, Jinzhong Liu, Yanrong Zou, Ping’an Peng

**Affiliations:** 1 State Key Laboratory of Organic Geochemistry, Guangzhou Institute of Geochemistry, Chinese Academy of Sciences, Wushan, Guangzhou 510640 China; 2 School of Environment and Energy, South China University of Technology, Guangzhou, 510006 China; 3 Dongguan Environmental Monitoring Centre Station, Dongguan, 523000 China; 4 University of Chinese Academy of Sciences, Beijing, 100049 China

**Keywords:** Solid Earth sciences, Geochemistry

## Abstract

Isotopic rollover refers to that δ^13^C value of a gas component decreases with maturity. Its occurrence is closely related to high productivity of shale gas. Isothermal confined pyrolysis experiments (gold capsules) were performed to simulate this phenomenon on whole rock Lucaogou and kerogens Saergan, Wuerhe and Fengcheng in the absence (anhydrous) and presence of added water (hydrous) at 50 MPa, 372 °C and heating duration 0–672 h, corresponding to 0.96–1.85 EASY%Ro. For kerogen Saergan isolated from source rock with hydrogen index (HI) 159 mg/g TOC and 1.10–1.30% Ro equivalent, none of δ^13^C_1_, δ^13^C_2_ and δ^13^C_3_ showed any rollover in both anhydrous and hydrous experiments. For Lucaogou whole rock with HI 856 mg/g TOC and 0.50–0.60%Ro, both δ^13^C_2_ and δ^13^C_3_ showed rollover in anhydrous experiments while all δ^13^C_1_, δ^13^C_2_ and δ^13^C_3_ showed rollover with greater magnitude in hydrous experiments starting at 1.49–1.64 EASY%Ro. For kerogens Wuerhe and Fengcheng isolated from source rocks with HI of 550 and 741 mg/g TOC, and 1.18 and 0.96%Ro respectively, both δ^13^C_2_ and δ^13^C_3_ demonstrated rollover in anhydrous experiments while only δ^13^C_2_ showed rollover with minor magnitude in hydrous experiments starting at 1.47–1.53 EASY%Ro. The different effects of water on isotopic rollover among samples Lucaogou, Wuerhe and Fengcheng can be ascribed to rate related isotopic fractionation. Higher generation rate leads to minor isotopic fractionation and rollover magnitude. It was suggested that isotopic rollover likely occurs in a source rock having higher amount of initial retained oil prior to bulk oil cracking and currently within the major stage of oil-cracking to gas (1.50–2.00%Ro).

## Introduction

Several studies have reported isotopic reversal and rollover of thermogenic gases, especially shale gases^1–7^. Isotopic reversal refers to that δ^13^C value decreases with carbon number of hydrocarbon gases. Isotopic rollover refers to that δ^13^C value of a gas component decreases with thermal stress levels^6,8^. Both are contrast to normal trends that δ^13^C value increases with maturity and carbon number of hydrocarbon gases observed in natural gas accumulations worldwide^9–13^. Reversal and rollover phenomena, especially the latter, have gained great attention because they are closely related to high productivity of shale gas^6,7,14^. To date, multiple interpretations have been proposed on these phenomena. Burruss and Laughrey^3^ suggested that Rayleigh fractionation during redox reactions creates isotopic reversals. Xia *et al*.^8^ demonstrated that mixing of primary and secondary gas in source rock can bring about isotopic reversal and rollover. Tilley and Muehlenbachs^6^ emphasized that gases from sealed, self-contained petroleum systems, including shale, very fine grained sandstone or siltstone, and some fractured, mixed clastic/carbonate reservoirs, have the maturation history different from conventional open system gases. The maturation history for gases in sealed, self-contained systems is comprised of three distinct stages, pre-rollover zone, rollover zone, and post-rollover zone^6^. Recently, Xia and Gao^15^ suggested that within the rollover zone, ethane and propane are decomposed and that the depletion of ^13^C in residual ethane and propane result from a partly reversal reaction scheme consisting of forward reaction, backward reaction and side reactions. Gao *et al*.^16^ performed a confined pyrolysis study on type II kerogen samples in the presence (hydrous) and absence of added water (anhydrous), and observed isotopic rollover for methane, ethane and CO_2_ in hydrous experiments, but did not observe the rollover for these components in anhydrous experiments.

Up to date, commercial shale gas has been produced from marine shales within the Lower Silurian Longmaxi Formation with maturity ranging %Ro 1.8–4.2 in southern Sichuan Basin, China^2^. Carbon isotopic reversals (δ^13^C_1_ > δ^13^C_2_ > δ^13^C_3_) were observed on shale gases produced from the Longmaxi Formation in this region^2^. However, the increase of δ^13^C_1_ and δ^13^C_2_ values with maturity demonstrates that these shale gases are in post-rollover zone^2,6^.

Previous studies demonstrated that the cracking of retained oil in source rocks is crucial for high production zone of shale gas^17,18^. In principle, high production zone of shale gas more likely occurs in a source rock having higher amount of initial retained oil prior to oil cracking and currently at maturity of major oil-cracking to gas. In natural system, a high amount of retained oil results from high amount of the generated oil or/and low oil expulsion efficiency in a source rock. The viewpoints in the previous studies by Tilley and Muehlenbachs^6^ and Xia *et al*.^8^ are closely related to the amount of initial retained oil, i.e. the close extent of sealed, self-contained petroleum system and the relative amount of secondary gas to primary gas.

In the present study, we aimed at the influence of the amount of the retained oil on the occurrence of isotopic rollover of gas components in source rocks, and performed confined pyrolysis experiments on four samples to simulate the isotopic rollover in laboratory. The proportion of gases derived from oil-cracking to kerogen-cracking is possibly the critical factor in controlling isotopic rollover of ethane and propane^6–8,17,18^. In order to examine the influence of water, as well as the amount of the retained oil in a source rock on isotopic reversal and rollover, three series of experiments were first carried out for a whole rock Lucaogou and a kerogen Saergan in anhydrous and hydrous experiments with water/TOC ratios of 2 and 4. Source rock Lucaogou has a low maturity (0.50–0.60%Ro) and high Rock-Eval hydrogen index (HI 856 mg HC/g TOC), representing a source rock with higher amount of retained oil, and subsequently higher proportion of gas derived from oil-cracking to kerogen-cracking. Kerogen Saergan has a high maturity (1.10–1.30%Ro equivalent) and low HI (159 mg/g TOC), representing a source rock with lower amount of retained oil and subsequently lower proportion of gas derived from oil-cracking to kerogen-cracking. Later, in order to evade multiple interpretations, such as mineral effect on the occurrence of isotopic rollover, we performed additional anhydrous and hydrous (water/TOC = 2) experiments on kerogens Wuerhe and Fengcheng to confirm the observations from the earlier experiments. These two kerogens have 1.18 and 0.96%Ro and HI 550 and 741 mg/g TOC, respectively representing source rocks with higher amount of retained oil and high proportion of gas derived from oil-cracking to kerogen-cracking.

## Samples and methods

### Samples

Four samples including one whole source rock (Lucaogou) and three kerogens (Saergan, Wuerhe and Fengcheng) were used in the present pyrolysis study. Source rock Lucaogou is a lacustrine mudstone collected from Middle Permian Lucaogou Formation (P_2l_) from borehole J23 in the southeastern area of the Junggar Basin. Kerogen Saergan was isolated from a marine mudstone collected from Middle-Upper Ordovician Saergan Formation (O_2–3s_) at Keping outcrop in the northwestern area of the Tarim Basin. Kerogen Wuerhe was isolated from a lacustrine mudstone within the Middle Permian Lower Wuerhe Formation (P_2w_) in borehole JT1 located at northwestern Junggar Basin. The Lower Wuerhe Formation (P_2w_) is an age-equivalent of the Lucaoguo Formation (P_2l_)^19^. Kerogen Fengcheng was separated from a tuffaceous dolomite within the Lower Permian Fengcheng Formation (P_1f_) in borehole F5 located at northwestern Junggar Basin^19^.

Whole rock Lucaogou has a high total organic carbon content (TOC 10.40 wt%) and HI (856 mg HC/g TOC). Therefore, whole rock Lucaogou (~200 mesh powder) without extraction was used directly in pyrolysis experiments. Whole rock Saergan has a relatively low TOC (1.81 wt%) and HI (159 mg HC/g TOC). Therefore, kerogen Saergan was used in pyrolysis experiments in order to obtain enough amounts of pyrolysates for chemical and isotopic analyses. Kerogens Wuerhe and Fengcheng were used in the later pyrolysis experiments to further confirm the observations of the earlier ones.

All the four source rock samples were ground to powder (~200 mesh). A major portion of Saergan, Wuerhe and Fengcheng powder samples and a minor portion of Lucaogou powder sample were then Soxhlet extracted with DCM:MeOH (93:7, v-v) for 72 h to remove bitumen. The extracted residues were treated with HCl and HF to obtain kerogens. Gross geochemical parameters for the four whole rocks and isolated kerogens are shown in Table 1.

**Table 1:** Gross geochemical parameters of whole rock and kerogen samples.

	Depth (m)	Strata	TOC %	%Ro	S1	S2	PI	HI	Tmax	C (%)	H (%)	O (%)	N (%)	δ^13^C (‰ PDB)
Lucaogou	2319.1	P_2l_	10.40	0.60	2.25	89.07	0.025	856	445	77.65	12.16	4.24	1.67	−28.71
Saergan	outcrop	O_2-3_	1.81	1.10–1.30	0.55	2.87	0.161	159	457	47.28	3.84	14.41	0.30	−30.10
Wuerhe	4660.6	P_2w_	3.37	1.18	0.41	18.52	0.02	550	450	56.04	7.93	3.70	0.67	−28.57
Fengcheng	3474.9	P_1f_	3.01	0.96	0.69	22.32	0.03	741	430	35.81	5.77	3.96	0.74	−27.12

TOC and Rock-Eval data were obtained from rock powder for all four samples. Rock-Eval parameters S1 and S2: in “mg HC/g rock”;

PI = S1/(S1 + S2); HI = S2/TOC, in “mg HC/g TOC”; Tmax: in “°C”; C (%), H (%), O (%), N (%) and δ^13^C (‰ PDB) were measured on kerogen

while the other data were measured on whole source rock.

### Confined pyrolysis experiments

Pyrolysis experiments on rock powder and kerogens were conducted in flexible gold capsules (4 mm o.d., 0.25 mm wall thickness and 60 mm length) contained within steel pressure vessels. The performance for these experiments is similar to that described in the previous studies^20–28^. Briefly, the capsules were welded at one end before being loaded with samples. Once loaded, the open end of each capsule was purged with argon, and then, if needed, deionized water was added which had justly been deoxygenated by bubbling with argon for 1 h. After water addition, the capsule was squeezed in a vise to create an initial seal, which was subsequently welded in the presence of argon. During welding, the previously welded end was immersed in cold water to prevent heating of reactants.

For the earlier experiments, six capsules containing whole rock Lucaogou and kerogen Saergan, each in anhydrous, and hydrous with water/TOC ratios of 2 and 4, respectively, were placed together in each vessel in order to maintain the same thermal conditions during heating. For the later experiments, the six capsules containing kerogens Wuerhe and Fengcheng, each in anhydrous and hydrous (water/TOC = 2), and a duplicate kerogen in anhydrous and hydrous (water/TOC = 2) were placed together in each vessel. Fourteen vessels were placed in a single furnace.

The vessels were connected to each other with tubing. The internal pressure of the vessels was preserved at 50 MPa within a variation range < ± 0.1 MPa by pumping water into or out of the vessels during the experiments.

The vessels containing gold capsules were heated to 250 °C within 10 h, and then from 250 °C to 372 °C at a rate of 2 °C/h. At 372 °C, the vessels were heated isothermally for 0–672 h, and then, removed from the oven (Table 2). Two thermocouples were used to detect the temperature and to cross-check. One thermocouple was attached to the outside wall of a specific vessel to detect the furnace temperatures. The other was placed inside this vessel to detect the temperatures of the gold tubes. The differences between the temperatures measured by the two thermocouples were <0.5 °C. The error of the measured temperatures is < ± 1 °C.

**Table 2:** EASY%Ro values versus heating time at 372 °C for LCG whole source rock and SEG, WEH and FC kerogens.

Time (h)	initial	0	24	48	72	96	144	192	240	288	336	384	432	480	528	576	624	672
Lucaogou	0.60	0.96	1.19	1.29	1.36	1.42	1.49	1.55	1.60	1.64	1.68	1.71	1.73	1.76	1.78	1.80	1.82	1.84
Saergan-1	1.10	1.16	1.28	1.35	1.40	1.45	1.52	1.57	1.62	1.65	1.69	1.72	1.74	1.77	1.79	1.81	1.83	1.85
Saergan-2	1.30	1.33	1.39	1.44	1.48	1.51	1.57	1.61	1.65	1.68	1.72	1.74	1.77	1.79	1.81	1.83	1.85	1.87
Wuerhe	1.18	1.21	—	1.37	—	1.47	1.53	1.58	1.63	1.66	1.69	1.72	—	1.77	—	1.82	—	1.85
Fengcheng	0.96	1.07	—	1.32	—	1.43	1.50	1.56	1.61	1.65	1.68	1.71	—	1.76	—	1.81	—	1.85

Time (h): duration at 372 °C; initial: %Ro value prior to heating. Saergan-1 and Saergan-2 are same sample with two different assumed initial Ro values.

### Chemical and carbon isotopic analyses of gas components

After pyrolysis, the volatile components in the capsules were collected in a special sampling device connected to a modified Agilent 6890 N GC, as described previously^20,24,26,28^. The gold capsule in the device, evacuated to <1 × 10^−2^ Pa, was pierced with a needle, allowing the gases to escape into the device. Analyses of both the organic and inorganic gas components were conducted by the GC from one injection in an automatically controlled procedure. For the hydrocarbon gas analysis, the oven temperature was initially held at 70 °C for 6 min, ramped from 70 to 130 °C at 15 °C/min, from 130 to 180 °C at 25 °C/min, and then held at 180 °C for 4 min. For the inorganic gas analysis, the oven temperature was held at 90 °C. The amounts of gas products measured using this device had a relative error <0.5%, tested using external standard gases.

After GC analysis, the remaining gas components entrapped within the device (about 80% of the initial amount) were taken for carbon isotopic analysis using a syringe to pierce the septum of the device, as described previously^20,24,26,28^. This analysis (GC-IRMS) was performed on a VG Isochrom II interfaced to an HP 5890 GC, fitted with a Poraplot Q column (30 m × 0.32 mm i.d.). Helium was used as the carrier gas. The column head pressure was 8.5 psi. The GC oven temperature was initially held at 40 °C for 3 min, ramped from 40 to 180 °C at 20 °C/min, and held at 180 °C for 5 min. A standard mixture of gaseous hydrocarbons (C_1_–C_4_) with known isotope compositions was used daily to test the performance of the instrument. The standard deviation for replicate analyses of this mixture is <0.3‰ for each compound^20,24,26,28^. For each capsule, isotopic analysis was performed two or three times. Deviations of δ^13^C values of each compound for repeated analyses were within 0.3‰. The averaged value for the two or three analyses was accepted.

## Results and discussion

### EASY%Ro for the capsules

Sweeney and Burnham^29^ present a vitrinite maturation model to calculate the vitrinite reflectance Ro%, using an Arrhenius first-order parallel-reaction approach with a distribution of activation energies (EASY%Ro). In the present study, EASY%Ro was used as a maturity parameter to indicate thermal stress achieved for the capsules at heating ramp from 250 °C to 372 °C at a rate of 2 °C/h, and isothermal stage at 372 °C for 0–672 h (Table 2). Heating duration of 0 h indicates at the end of heating ramp. The initial maturity is low for Lucaogou sample within 0.50–0.60%Ro^30^. EASY%Ro of Lucaogou whole rock ranges 0.96–1.84 from heating time 0–672 h at 372 °C. Kerogen Saergan was isolated from Ordovician source rock having no vitrinite. The initial maturity for this kerogen was estimated at 1.10–1.30%Ro equivalent based on reflectance of pyrobitumen, Rock-Eval Tmax, content of extracted bitumen and molecular parameters^31^. We suppose that kerogen Saergan had suffered a heating process prior to present pyrolysis experiment, e.g., from 250 °C to 386 °C or 404 °C at 2 °C/h, achieving a thermal stress of 1.10 or 1.30 EASY%Ro. Therefore, EASY%Ro of kerogen Saergan ranges 1.16–1.85 or 1.33–1.87 from heating time 0–672 h at 372 °C (Table 2). In this paper, only the range of 1.16–1.85 EASY%Ro for kerogen Saergan was used. Similarly, the calculated EASY%Ro for kerogens Wuerhe and Fengcheng are in the ranges of 1.21–1.85 and 1.07–1.85 (Table 2).

### Generation and cracking of gaseous hydrocarbons

**Figure 1:**
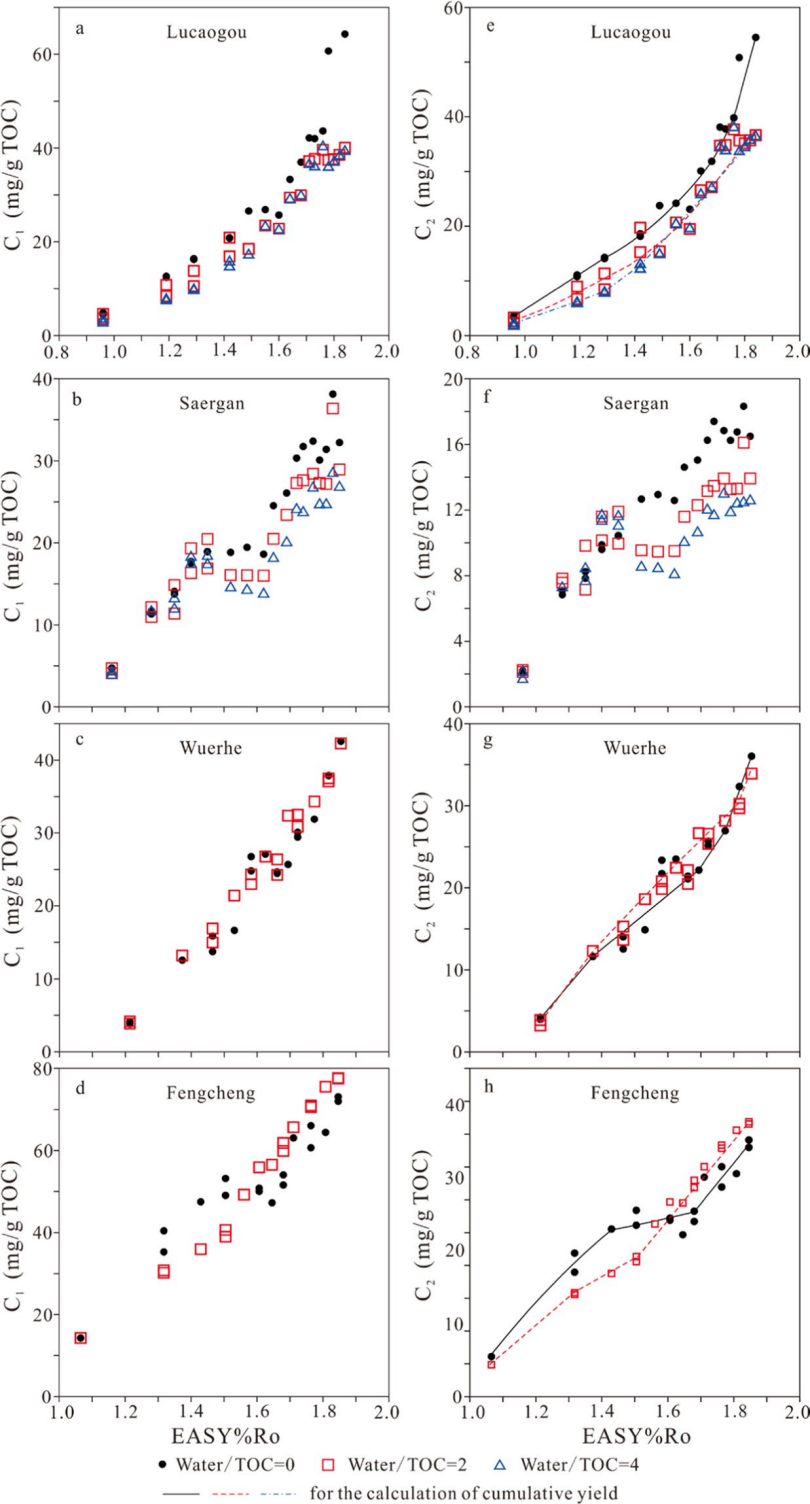
The amounts of generated methane and ethane versus EASY%Ro.

**Figure 2:**
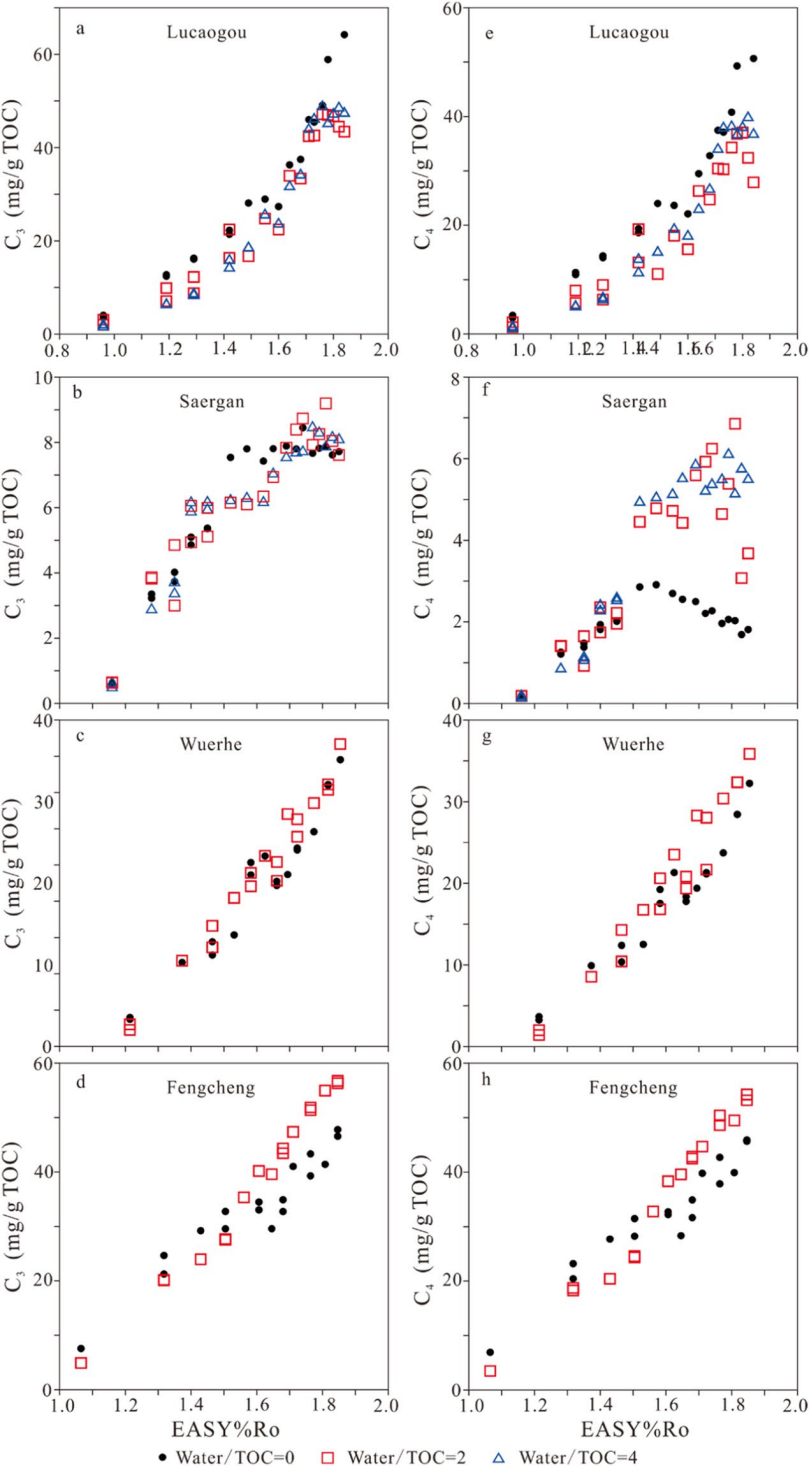
The amounts of generated propane and butanes (*i*- + *n*-butanes) versus EASY%Ro.

**Figure 3:**
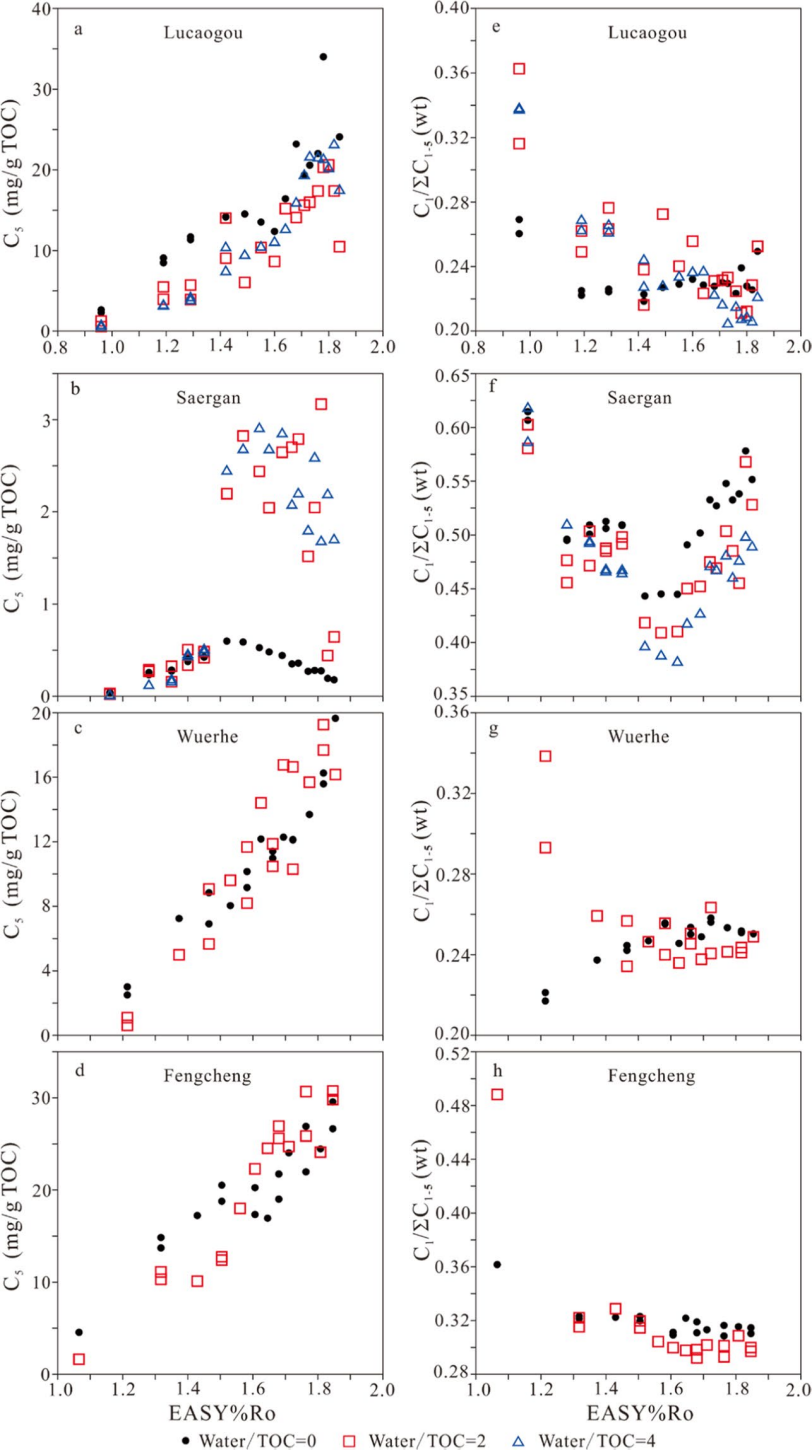
The amounts of generated pentanes (*i*- + *n-*pentanes) and dryness ratio (C_1_/ΣC_1–5_) versus EASY%Ro.

For whole rock Lucaogou, the yields of methane, ethane, propane, butanes and pentanes generally increase with EASY%Ro and are relatively higher in anhydrous experiments than hydrous experiments (Figs. 1a,e, 2a,e, and 3a). Yield decrease of butanes and pentanes for hydrous experiments at 1.76–1.84 EASY%Ro can be mainly ascribed to sample heterogeneities (Figs. 2e and 3a). Otherwise, if we ascribe this decrease trend to butane and pentane decomposition the yields of methane and ethane would increase noticeably, contrast to the observed result (Fig. 1a,e).

For kerogen Saergan, the yields of C_1_ to C_3_ increase consistently with EASY%Ro while the yields of C_4_ and C_5_ have a clear decreasing trend at high maturity (Figs. 1b,f, 2b,f and 3b). The yields of all gaseous hydrocarbons (C_1_ to C_5_) are similar at EASY%Ro <1.40–1.52 but differ significantly at EASY%Ro >1.40–1.52 between anhydrous and hydrous experiments (Figs. 1b,f, 2b,f and 3b). The yields of butanes and pentanes in anhydrous experiments reached the maximum values at 1.57 EASY%Ro, and are substantially lower than those in hydrous experiments at >1.57 EASY%Ro (Figs. 2f and 3b). This result demonstrates that butanes and pentanes were less thermally stable and decomposed earlier to form smaller molecules, leading to that methane and ethane yields are significantly higher at >1.57 EASY%Ro in anhydrous experiments than hydrous experiments (Fig. 1b,f).

For kerogens Wuerhe and Fengcheng, the yields of gaseous hydrocarbons (C_1_ to C_5_) increase consistently with EASY%Ro in both anhydrous and hydrous experiments (Figs. 1c–d,g–h, 2c–d,g–h, and 3c–d). For kerogen Wuerhe, the yields of these components in hydrous experiments are similar to, or slightly higher than those in anhydrous experiments (Figs. 1c,g, 2c,g, and 3c). For kerogen Fengcheng, the yields of these gases are clearly lower in hydrous experiments than anhydrous experiments at ≤1.50 EASY%Ro while it is opposite at ≥1.61 EASY%Ro (Figs. 1d,h, 2d,h, and 3d).

Previous studies demonstrated that water has twofold influences on hydrocarbon generation in pyrolysis experiments: (1) water reduces organic maturation rate, including the rates of both hydrocarbon generation and cracking^21–23,32,33^; and (2) hydrogen and oxygen from water can be incorporated into the generation of hydrocarbon and CO_2_^21,33,34^. For whole rock Lucaogou, generation rates of gaseous hydrocarbons are lower in hydrous experiments than anhydrous experiments (Figs. 1a,e, 2a,e, and 3a). For kerogen Saergan, butanes and pentanes are more thermally stable in hydrous experiments than anhydrous experiments (Figs. 2f and 3b). Both phenomena can be ascribed to water retardation effect. For kerogen Wuerhe, water had no clear influence on gas generation (Figs. 1c,g, 2c,g, and 3c). For kerogen Fengcheng, water retarded gas generation at ≤1.50 EASY%Ro but accelerated gas generation at ≥1.61 EASY%Ro (Figs. 1d,h, 2d,h, and 3d). The cause behind this phenomenon is unknown to us.

Previous studies demonstrated that the maturities for the maximum yields of C_2_, C_3_, C_4_ and C_5_ are in the range of 2.2–3.2, 1.8–2.4, 1.6–2.3 and 1.5–1.8 EASY%Ro, respectively, in confined pyrolysis experiments on type I lacustrine kerogens and marine oil while they are in the range of 1.7–2.0, 1.5–1.7, 1.4–1.7 and 1.3–1.6 EASY%Ro, respectively, in confined pyrolysis experiments on extracted coal, coal and coal plus bitumen^24–26,30^. When the yields of wet gases reach maximum values, the generation rates equal the removal rates by cracking of these components. For the experiments on whole rock Lucaogou and kerogens Wuerhe and Fengcheng, the yields of wet gases (C_2_ to C_5_) do not show any clear decreasing trend, and have not reached the maximum values (Figs. 1–3). For kerogen Saergan experiments, the yields of C_2_ and C_3_ do not show any clear decreasing trend and have not reached the maximum values but the yields of C_4_ and C_5_ show a clear decreasing trend and have achieved the maximum values (Figs. 1f, 2b,f and 3b). Previous studies demonstrated that solid organic matter accelerates hydrocarbon cracking^25–28,35–37^. A higher amount of oil relative to total organic carbon reduces kerogen catalytic effect on the cracking of oil and wet gases^25,26^. The different trends of C_4_ and C_5_ yields between kerogen Saergan and the other three samples can be primarily ascribed to oil/TOC ratio in the reaction cells. In summary, for the experiments of all whole rock and kerogens, wet gases, especially ethane and propane, did not crack prominently at <1.85 EASY%Ro.

### Dryness ratio (C_1_/ΣC_1–5_)

For whole rock Lucaogou, in anhydrous experiments, dryness ratio (C_1_/ΣC_1–5_) decreased at 0.96–1.19 EASY%Ro, and remained unchanged at 1.19–1.84 EASY%Ro. However, in hydrous experiments, this ratio decreased consistently at 0.96–1.84 EASY%Ro (Fig. 3e). For kerogen Saergan, in both anhydrous and hydrous experiments, C_1_/ΣC_1–5_ ratio decreased to the lowest value at 1.52–1.62 EASY%Ro, and then increased (Fig. 3f). For kerogen Wuerhe, C_1_/ΣC_1–5_ ratio increased in anhydrous experiments, but decreased in hydrous experiments at ≤1.47 EASY%Ro, and then, remained stable in both cases at ≥1.53 EASY%Ro (Fig. 3g). For kerogen Fengcheng, C_1_/ΣC_1–5_ ratio decreased rapidly at maturities from 1.07 to 1.32 EASY%Ro, and then remained stable with maturity in both anhydrous and hydrous experiments (Fig. 3h).

Previous studies demonstrated that dryness ratio (C^1^/ΣC_1–5_) at first decreases, and then increases with thermal maturity in pyrolysis experiments on kerogen and source rock samples^21,26,38,39^. In the studies by Pan *et al*.^21^ and Li *et al*.^26^, C_1_/ΣC_1–5_ ratio demonstrated a negative correlation with the yield of liquid alkanes (ΣC_8+_), reaching the minimum value at the maturity for the maximum ΣC_8+_. Evans and Felbeck^38^ observed that C_1_/ΣC_1–4_ ratio maintained the minimum value at temperature interval 420–440 °C and heating time 16 h, equivalent to EASY%Ro 1.68–2.00 in pyrolysis experiments on Green River Shale containing Type I kerogen. Lorant *et al*.^39^ found that C_1_/ΣC_1–5_ ratio maintained the minimum value at temperature 350–400 °C and heating duration 24 h, equivalent to EASY%Ro 0.93–1.49 in pyrolysis experiments on a Type II kerogen. In the present study, for kerogen Saergan, liquid alkanes decomposed rapidly at EASY%Ro > ~1.50, as demonstrated by the trend of pentane yield (Fig. 3b), leading to an increasing trend of C_1_/ΣC_1–5_ ratio (Fig. 3f). For whole rock Lucaogou and kerogens Wuerhe and Fengcheng with high oil potentials (HI), the amounts of liquid alkanes, in particular light alkanes remained very high at EASY%Ro 1.20–1.85, referenced from the trend of pentane yield (Fig. 3a,c–d), and therefore, C_1_/ΣC_1–5_ ratio maintained the minimum value within this maturity interval (Fig. 3e,g–h).

Gao *et al*.^16^ observed that the amount of methane relative to other hydrocarbon gases increased due to water addition and suggested that water inhibited the generation of C_2+_ from kerogen, and/or promoted the secondary cracking of C_2+_ hydrocarbons to methane. In the present study, C_1_/ΣC_1–5_ ratio increased for whole rock Lucaogou but it decreased for kerogen Saergan due to water addition (Fig. 3e,f). For kerogens Wuerhe and Fengcheng experiments, C_1_/ΣC_1–5_ ratio increased at lower EASY%Ro but it decreased at higher EASY%Ro due to water addition (Fig. 3g,h). It appears that the influence of water addition on C_1_/ΣC_1–5_ ratio is complicated.

### Carbon isotopic rollover and reversal

**Figure 4:**
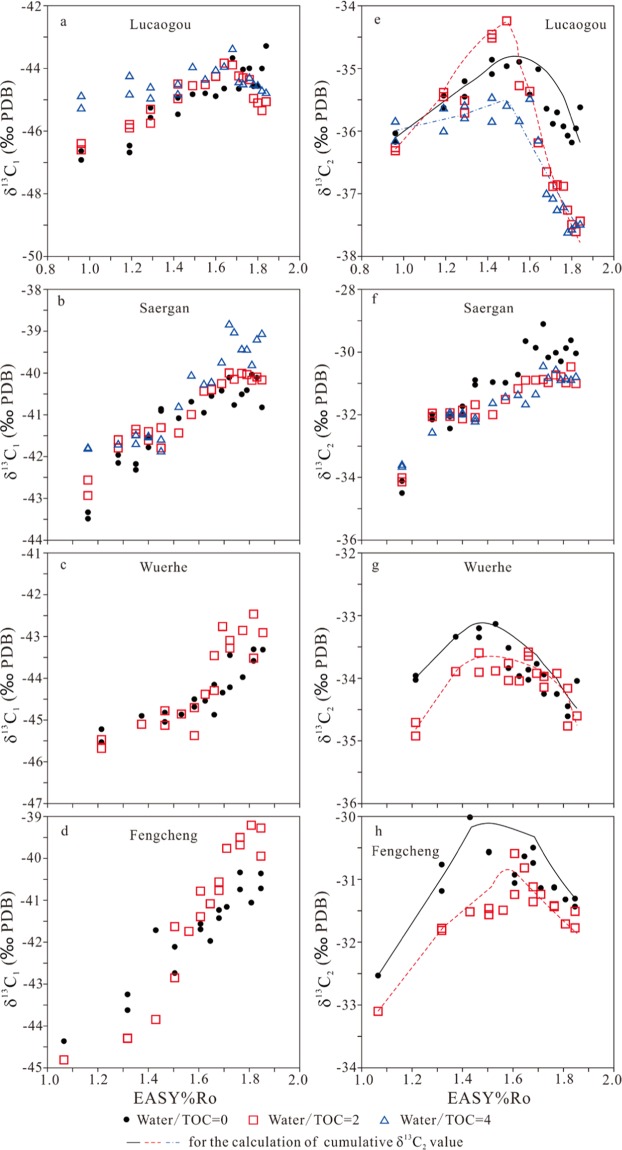
Carbon isotopes of generated methane (δ^13^C_1_) and ethane (δ^13^C_2_) versus EASY%Ro.

**Figure 5:**
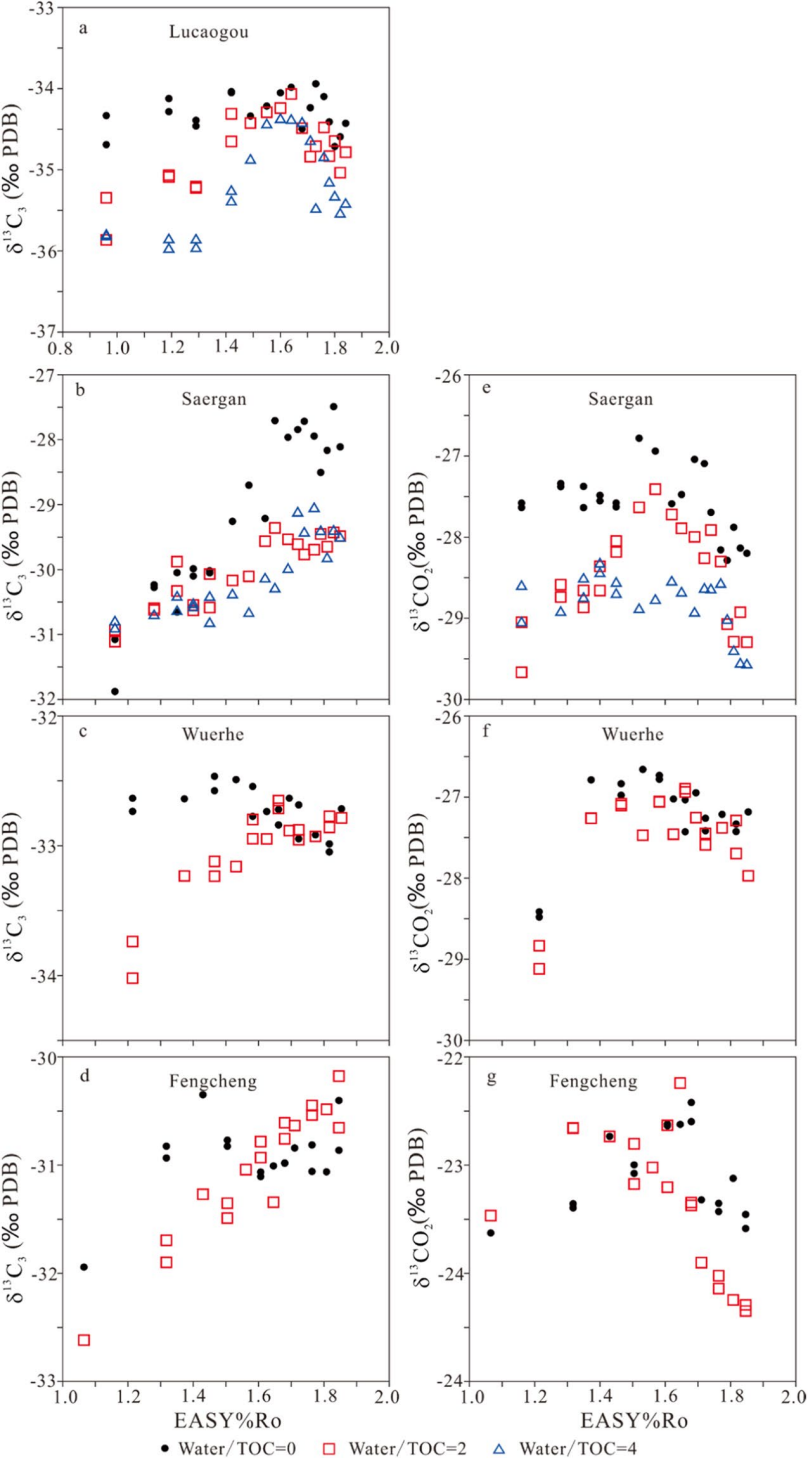
Carbon isotopes of generated propane (δ^13^C_3_) and CO_2_ (δ^13^CO_2_) versus EASY%Ro.

Carbon isotopic compositions of methane, ethane, propane and carbon dioxide for the experiments of the four samples are shown in Figs. 4 and 5.

In anhydrous experiments, for whole rock Lucaogou, carbon isotopes of methane (δ^13^C_1_) did not demonstrate any rollover but carbon isotopes of ethane (δ^13^C_2_) and propane (δ^13^C_3_) clearly demonstrated rollover starting at 1.55 and 1.64 EASY%Ro, respectively (Figs. 4a,e and 5a). For kerogen Saergan, none of δ^13^C_1_, δ^13^C_2_ and δ^13^C_3_ demonstrated any rollover (Figs. 4b,f and 5b), but δ^13^C of CO_2_ demonstrated a clear rollover starting at 1.52 EASY%Ro (Fig. 5e). For kerogen Wuerhe, δ^13^C_1_ did not demonstrate any rollover but both δ^13^C_2_ and δ^13^C_3_ clearly demonstrated a rollover starting at 1.53 EASY%Ro (Figs. 4c,g and 5c). The rollover extent is relatively higher for δ^13^C_2_ than δ^13^C_3_ (Figs. 4g and 5c). δ^13^C of CO_2_ demonstrated a clear rollover starting at 1.53 EASY%Ro (Fig. 5f). For kerogen Fengcheng, the variation trends of δ^13^C_1_, δ^13^C_2_, δ^13^C_3_ and δ^13^C of CO_2_ are similar to those for kerogen Wuerhe (Figs. 4d,h and 5d,g).

In hydrous experiments, for whole rock Lucaogou, all of δ^13^C_1_, δ^13^C_2_ and δ^13^C_3_ demonstrated rollover starting at 1.49–1.64 EASY%Ro (Figs. 4a,e and 5a). For kerogen Saergan, none of δ^13^C_1_, δ^13^C_2_ and δ^13^C_3_ demonstrated any rollover (Figs. 4b,f, and 5b), but δ^13^C of CO_2_ demonstrated a clear rollover starting at 1.57–1.77 EASY%Ro (Fig. 5e). For kerogen Wuerhe, neither δ^13^C_1_ nor δ^13^C_3_ demonstrated any rollover, but δ^13^C_2_ clearly demonstrated a rollover starting at 1.47 EASY%Ro (Figs. 4c,g and 5c). δ^13^C of CO_2_ demonstrated a clear rollover starting at 1.66 EASY%Ro (Fig. 5f). For kerogen Fengcheng, the variation trends of δ^13^C_1_, δ^13^C_2_, δ^13^C_3_ and δ^13^C of CO_2_ are similar to those for kerogen Wuerhe (Figs. 4d,h and 5d,g).

**Figure 6:**
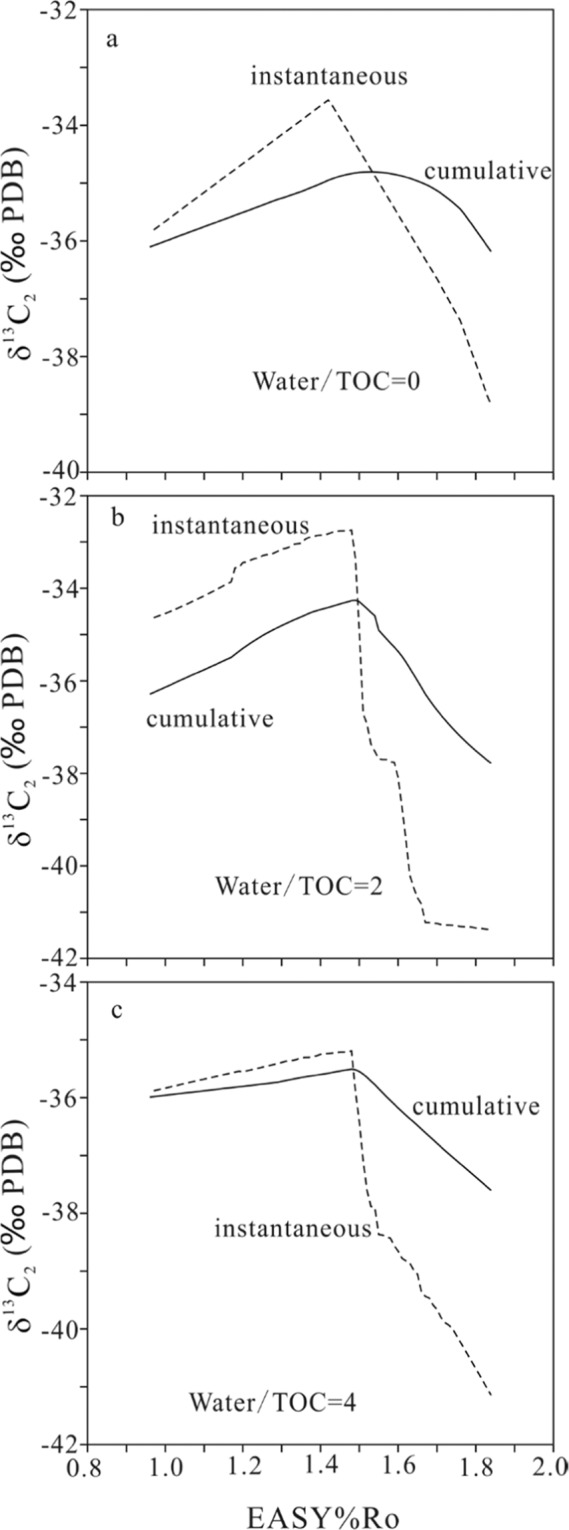
Cumulative and instantaneous carbon isotopes of generated ethane (δ^13^C_2_) versus EASY%Ro for whole rock Lucaogou. Instantaneous carbon isotopes were calculated within a maturity interval of EASY%Ro 0.10.

**Figure 7:**
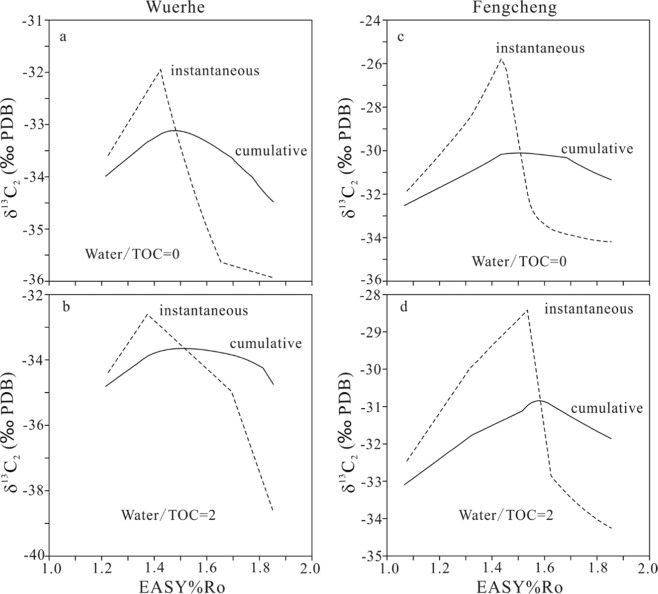
Cumulative and instantaneous carbon isotopes of generated ethane (δ^13^C_2_) versus EASY%Ro for keogens Wuerhe (**a,b**) and Fengcheng (**c,d**). Instantaneous carbon isotopes were calculated within a maturity interval of EASY%Ro 0.10.

In the confined pyrolysis experiments, the measured yields and carbon isotopes for gas components are cumulative (Figs. 1–5). Ethane has the most prominent isotopic rollover among C_1_–C_3_ gases for samples Lucaogou, Wuerhe and Fengcheng. Therefore, the instantaneous carbon isotopes for ethane (δ^13^C_2_) within a maturity interval of 0.10 EASY%Ro were calculated based on the cumulative yields and carbon isotopes for the three samples (Figs. 6 and 7). The rollover extents are substantially greater for the calculated instantaneous than the measured cumulative δ^13^C_2_ values (Table 3, Figs. 6–7). However, the rollover extents are not proportionally related between the instantaneous and cumulative values (Table 3, Figs. 6–7).

**Table 3:** Rollover extents for carbon isotopes (δ^13^C ‰ PDB) of gas components.

Samples	Lucaogou	Lucaogou	Lucaogou	Saergan	Saergan	Saergan	Wuerhe	Wuerhe	Fengcheng	Fengcheng
Water/TOC	0	2	4	0	2	4	0	2	0	2
δ^13^C_1_ C	N	1.5‰	1.4‰	N	N	N	N	N	N	N
δ^13^C_2_ C	1.3‰	3.3‰	2.2‰	N	N	N	1.5‰	1.0‰	1.4‰	1.2‰
δ^13^C_2_ I	5.3‰	8.6‰	6.0‰	—	—	—	4.0‰	6.1‰	8.4‰	5.8‰
δ^13^C_3_ C	0.7‰	1.0‰	1.2‰	N	N	N	0.6‰	N	0.7‰	N
δ^13^C_CO2_ C	—	—	—	1.5‰	1.9‰	1.2‰	0.8‰	1.0‰	1.2‰	2.1‰

C: cumulative; Ι: instantaneous within a maturity interval of EASY%Ro 0.10; N: no clear rollover;—: not measured or calculated.

**Figure 8:**
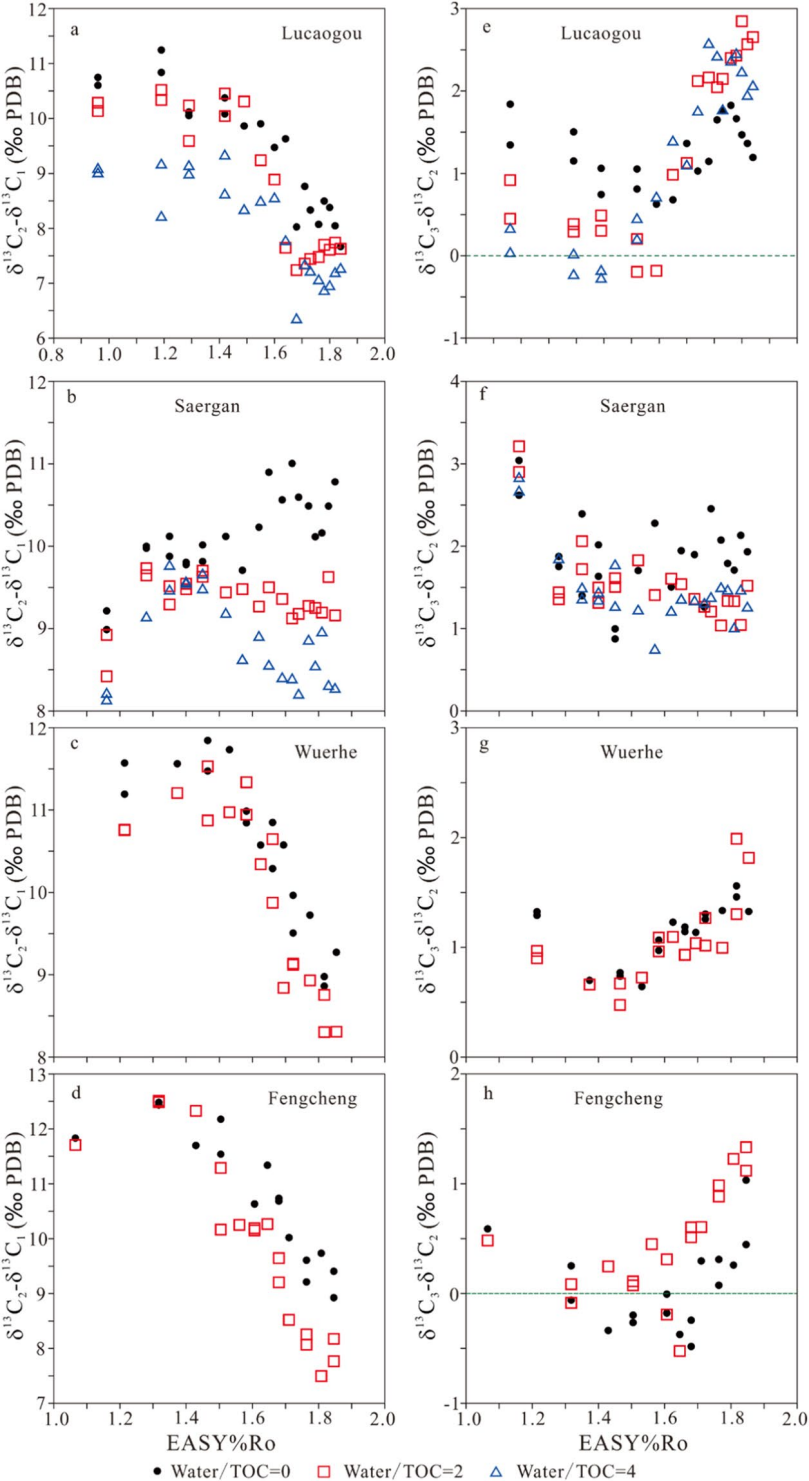
Parameters δ^13^C_2_–δ^13^C_1_ and δ^13^C_3_–δ^13^C_2_ versus EASY%Ro.

For four samples, δ^13^C_1_ are substantially more negative than δ^13^C_2_ with a difference in the range of 6–13‰ (Fig. 8a–d). However, parameter δ^13^C_2_–δ^13^C_1_ has a clear decrease trend with maturity starting at 1.32–1.42 EASY%Ro for whole rock Lucaogou and kerogens Wuerhe and Fengcheng in both anhydrous and hydrous experiments (Fig. 8a,c,d). This trend can be also observed for kerogen Saergan in hydrous experiments but it cannot be observed in anhydrous experiments (Fig. 8b).

For whole rock Lucaogou and kerogens Wuerhe and Fengcheng, parameter δ^13^C_3_–δ^13^C_2_ firstly decreases to the lowest values at 1.40–1.60 EASY%Ro, and then increases, having a clear reversal trend (Fig. 8e,g,h). For whole rock Lucaogou and kerogen Fengcheng, this parameter has the lowest value <0, indicating a reversal between δ^13^C_2_ and δ^13^C_3_ (Fig. 8e,h). For kerogen Wuerhe, this parameter has the lowest value > 0. For kerogen Saergan, this parameter at first decreases, and then remains stable with EASY%Ro (Fig. 8f).

### Mechanism for carbon isotopic rollover and implications

Tian *et al*.^40^ performed a confined pyrolysis study on whole oil and fractions separated from the oil and demonstrated that δ^13^C_1_, δ^13^C_2_ and δ^13^C_3_ values increase from saturated fraction, whole oil, asphaltene fraction to aromatic fraction. These values for saturated fraction are significantly lower than for fractions of asphaltenes and aromatics with a difference up to, or even higher than 10‰ from 375 °C to 480 °C (~1.80 EASY%Ro) at 20 °C/h, and from 375 °C to 450 °C (~1.98 EASY%Ro) at 2 °C/h. Although δ^13^C_1_ values differ substantially at lower temperature and maturity, they are very similar at the final temperature and maturity (4.46 EASY%Ro) among the whole oil and fractions of saturates, aromatics and asphaltenes at both heating rates. This result demonstrated that carbon isotopes of the precursor for methane generation are similar among the whole oil and oil fractions, and the difference in carbon isotopes of gaseous hydrocarbons at early cracking stage can be mainly ascribed to differential isotopic fractionation effect (ΔEa value) among whole oil and oil fractions during gas generation^13,41^. Similar result was also obtained in the study by Li *et al*.^26^.

Tilley and Muehlenbachs^6^ and Xia *et al*.^8^ divided carbon isotopic variation trend into three stages with increasing maturity of gaseous hydrocarbons generated from a single source rock: (1) pre-rollover zone, δ^13^C values of gaseous hydrocarbons increase with maturity; (2) rollover zone, δ^13^C_2_ and δ^13^C_3_ values become more negative with maturity; and (3) post rollover zone, δ^13^C values of gaseous hydrocarbons increase with maturity, back to a normal variation trend.

At pre-rollover zone with low maturity (<1.50%Ro), kerogen and polar components of oil are less thermally stable and preferentially crack^42,43^, generating gaseous hydrocarbons with relatively heavy carbon isotopes^8,13,40,41^, while saturated components are more thermally stable, generating no or very little gaseous hydrocarbons^8^. At rollover zone with bulk generation of gaseous hydrocarbons (1.50–2.00%Ro)^6–8^, gaseous hydrocarbons are mainly generated from the cracking of saturated components and have lighter carbon isotopes. At post rollover zone (%Ro > 2.00)^2,6^, residual saturated components are increasingly rich in ^13^C and wet gases themselves begin to crack. Both causes lead to carbon isotopes of wet gases increasingly heavier with maturity.

As discussed above, the amount of saturated components relative to polar components and kerogen is critical to the variation trend of δ^13^C values for gaseous hydrocarbons in pre-rollover and rollover zones within a source rock. The mixing of gaseous hydrocarbons from the cracking of kerogen and polar components with those from the cracking of saturated components results in the carbon isotopic rollover of wet gases^8,40^. The maturity for the experiments in the present study is in the range from the late pre-rollover to middle rollover zone. For experiments on whole rock Lucaogou and kerogens Wuerhe and Fengcheng with high HI values, the amount of the generated oil or saturated components relative to kerogen and polar components is very high, therefore, carbon isotopic rollover is clearly demonstrated. The initial kerogen Saergan possibly had high oil generative potential or HI value at immature stage, but it passed the peak oil generation stage in geologic history. This kerogen currently has high maturity and low HI value (Table 1), and can only generate a limited amount of oil in experiments. The amount of gaseous hydrocarbons generated from the cracking of the generated oil is too low, and therefore, carbon isotopic rollover for gaseous hydrocarbons was not observed in experiments.

In natural source rocks, the amount of the retained oil is controlled by both the amount of the generated oil and oil expulsion efficiency. Oil expulsion results in substantial fractionation of bulk oil composition. The expelled oil contains higher amount of saturated fraction and lower amounts of resin and asphaltene fractions, compared with the residual oil in the source rock^44^. For an oil prone source rock with high oil expulsion efficiency, only a small amount of residual oil is retained, which is dominated by polar components due to major compositional fractionation, and mainly absorbed by kerogen. The amount of saturated components relative to polar components and kerogen is very low for this source rock. In contrast, for an oil-prone source rock with low oil expulsion efficiency, a significantly high amount of residual oil is retained, which contains relatively high amount of saturated components due to minor compositional fractionation. The residual oil can be absorbed by kerogen, and in micro fractures within the source rock and sandstone or porous rock layers with small thickness surrounded by the source rock. The amount of saturated components relative to polar components and kerogen is considerably high in this source rock. The higher amount of the retained oil leads to higher amount of shale gas in bulk oil-cracking stage. This result of the present experiments is consistent to the observation that the isotopic rollover is closely related to the high productivity of shale gas^6,7,14^.

Zumberge *et al*.^7^ found that carbon isotopic rollover for shale gas started at ~1.50%Ro, and suggested that carbon isotopic data of gaseous hydrocarbons can be used as maturity indicator for shale gas and source rocks. The result of the present study is consistent to the observation by Zumberge *et al*.^7^. The variation trend for carbon isotopes of wet gases with maturity, such as pre-rollover, rollover and post rollover zones, can be used as a guideline for using carbon isotopes of gaseous hydrocarbons as thermal maturity indicator for source rocks with higher amount of the initial retained oil. However, isotopic rollover zone may not be observed in a source rock with lower amount of the initial retained oil as demonstrated in the experiments on kerogen Saergan in the present study.

The measured cumulative carbon isotopes have rollover shifts lower than 3.5‰ for C_1_–C_3_ gases, and in the range of 1.0–3.5‰ for C_2_ in the experiments of whole rock Lucaogou and kerogens Wuerhe and Fengcheng. However, the calculated instantaneous carbon isotopes within a maturity interval of 0.10 EASY%Ro have rollover shifts in the range of 4.0–8.6‰ for C_2_ for the three samples (Table 3, Figs. 6 and 7), closer to 8–10‰ in the natural system reported by Zumberge *et al*.^7^, compared with the measured cumulative carbon isotopes. Xia^45^ suggested that shale gas within the Barnett Shale is close to instantaneous based on the variation trend of wetness with maturity. Smaller isotopic rollover shift in pyrolysis experiments compared with in nature can be ascribed to temperature difference. High temperature led to less isotopic fractionation in the formation of gaseous hydrocarbons in experiments^13,41^.

Water effects on carbon isotopic rollover of methane, ethane and propane are different for different samples (Table 3 and Figs. 4–7). In whole rock Lucaogou experiments, water substantially increased the rollover magnitude for both cumulative and instantaneous δ^13^C_2_ (Table 3, Fig. 6). In kerogen Wuerhe experiments, water decreased rollover magnitude of cumulative δ^13^C_2_ but increased rollover magnitude of instantaneous δ^13^C_2_ (Table 3, Fig. 7a,b). In kerogen Fengcheng experiments, water decreased the rollover magnitude for both cumulative and instantaneous δ^13^C_2_ (Table 3, Fig. 7c,d). Gao *et al*.^16^ observed that isotopic rollover for methane, ethane and CO_2_ occurred in hydrous experiments, but did not occur in anhydrous experiments, and suggested that water involved reaction is responsible for the isotopic rollover. Some other researchers held similar point of view^3,7^. Here, we present an alternative or additional interpretation on water effect: water influences the generation rates of gaseous hydrocarbons and consequently influences isotopic fractionation between precursor and gas products. The reaction rate dependent isotopic fractionation is similar to temperature dependent isotopic fractionation. Higher reaction rate leads to lower isotopic fractionation^20^. In the experiments of whole rock Lucaogou, generation rates of gaseous hydrocarbons significantly reduced due to water addition (Figs. 1–3), leading to greater rollover magnitude for both cumulative and instantaneous carbon isotopes (Table 3, Figs. 4–6). In the experiments of kerogens Wuerhe, generation rates of gaseous hydrocarbons were similar or changed slightly due to water addition (Figs. 1–3), leading to different effects on rollover magnitude between cumulative and instantaneous carbon isotopies (Table 3, Fig. 7a,b). In kerogen Fengcheng experiments, generation rates of gaseous hydrocarbons decreased at EASY%Ro <1.4 but increased significantly at EASY%Ro > 1.4 due to water addition (Figs. 1–3), leading to smaller rollover magnitude for both cumulative and instantaneous carbon isotopes (Table 3, Fig. 7c,d). In the study by Gao *et al*.^16^, the yields of gaseous hydrocarbons from kerogens decreased due to water addition, therefore, carbon isotopic rollover magnitude increased for gas components, similar to our experiments on whole rock Lucaogou. It can be expected that whole rock Lucaogou in hydrous experiments could be closer to natural source rocks compared with kerogen samples. Therefore, greater isotopic rollover extents were demonstrated from field data^7^.

For whole rock Lucaogou, carbon isotopic rollover for methane occurred in hydrous experiments but not in anhydrous experiments, consistent with the observation by Gao *et al*.^16^. However, carbon isotopic rollover for methane did not occur in both hydrous and anhydrous experiments on all the three kerogens (Fig. 4b–d). Furthermore, Zumberge *et al*.^7^ observed carbon isotopic rollover for ethane, propane and CO_2_, but not for methane in nature. It appears that water influence on carbon isotopic rollover of methane is effective only in laboratory condition, not in natural system.

According to the results of our experimental study, in combination with field observations^2,3,5–7^, we suggest that the following two factors are important for isotopic rollover and reversal in a source rock: (1) higher amount of initial retained oil prior to bulk oil cracking, and (2) currently within the major stage of oil-cracking to gas (1.50–2.00%Ro). The first factor leads to higher amount of saturated components relative to kerogen and polar components. The second factor is the maturity range for rollover zone. Xia and Gao^15^ presented a partly reversible reaction scheme with a forward, a backward and a side reaction to investigate the isotopic fractionation, and provided a better interpretation on the rollover of carbon isotopes for ethane and propane within the Barnett Shale. We do not exclude this reaction mechanism, and believe that this reaction mechanism could be a supplementary for the mixing model of kerogen-cracking gas with oil-cracking gas presented in the previous study^8^.

Prior to the study by Gao *et al*.^16^ documenting the isotopic rollover during laboratory experiments, numerous studies had been reported on carbon isotopic compositions of gaseous hydrocarbons in pyrolysis experiments on oil and kerogen^24,39,40,46^. In the study by Hill *et al*.^46^ on oil anhydrous experiments in confined system, rollover for δ^13^C_2_ can be clearly observed from five experiments at 360 °C and heating times 3, 6, 12, 24 and 33.3 days, corresponding to 1.13, 1.29, 1.44, 1.59 and 1.67 EASY%Ro, respectively. Values of δ^13^C_2_ for the five experiments are −38.79, −38.78, −38.87, −39.25 and −39.53‰, demonstrating a clear rollover starting at 1.29 EASY%Ro with the shift up to 0.75‰. However, δ^13^C values for methane and propane vary irregularly with heating time for these five experiments^46^. In the other previous studies aforementioned, isotopic rollover cannot be observed. These results can be ascribed to the following causes:

1. In programing heating experiments, different maturities were achieved at different temperatures, leading to different extents of isotopic fractionation between the generated gas components and their precursors^13,41^. In natural system, the heating rate is generally within 1–5 °C/Myr, much lower that at laboratory experiments, i.e., 2 and 20 °C/h.
2. Lorant *et al*.^39^ performed isothermal experiments using different temperatures from 275–555 °C to achieve different maturities, so that carbon isotopes of gas components did not show rollover trend with maturity and temperatures. Similarly, Hill *et al*.^46^ conducted isothermal experiments using temperatures from 350–450 °C to achieve different maturities from EASY%Ro 1.02–2.83. In the study by Hill *et al*.^46^, δ^13^C_2_ data vary irregularly without any rollover trend for all experiments but they show a clear rollover for five experiments at 360 °C as mentioned earlier.
3. Xia and Gao^15^ suggested that temperatures lower than 400 °C are favorable for the backward reaction, leading to isotopic rollover. At higher temperatures, the backward reaction is suppressed or the side reaction is enhanced, and no ^13^C depletion is observed during ethane and propane decomposition^15^.

## Conclusions

In both anhydrous and hydrous pyrolysis experiments, carbon isotopic rollover of gas components occurred for whole rock Lucaogou and kerogens Wuerhe and Fengcheng with higher HI ranging 550–856 mg HC/g TOC but did not occur for kerogen Saergan with lower HI of 159 mg/g TOC, demonstrating that the amount of the generated oil or the proportion of gas components derived from oil cracking to kerogen cracking could be the critical factor controlling this rollover phenomenon. The different effects of water on isotopic rollover among samples Lucaogou, Wuerher and Fengcheng can be ascribed to rate related isotopic fractionation. Higher generation rate of gas components leads to minor isotopic fractionation and rollover magnitude.

## Supplementary information

Supplementary information.
