# Supplementary material for: Isothermal confined pyrolysis on source rock and kerogens in the presence and absence of water: Implication in isotopic rollover in shale gases
Source: Sci Rep. 2020 Mar 31;10:5721. doi: 10.1038/s41598-020-62790-6 (PMC7109094; doi:10.1038/s41598-020-62790-6)
Supplement: Supplementary file 1 — Supplementary information. [file 41598_2020_62790_MOESM1_ESM.pdf]

Isothermal confined pyrolysis on source rock and kerogens in the presence and absence of water: Implication in isotopic rollover in shale gases

Hao Xu [1,2,3], Changchun Pan [1]*, Lifei Zeng [1,4], Wenkui Huang [1,4], Chenxi Zhou [1,4], Shuang Yu [1], Jinzhong Liu [1], Yanrong Zou [1], Ping'an Peng [1]

[1] *State Key Laboratory of Organic Geochemistry, Guangzhou Institute of Geochemistry, Chinese Academy of Sciences, Wushan, Guangzhou 510640, China*

[2] *School of Environment and Energy, South China University of Technology, Guangzhou 510006, China*

[3] *Dongguan Environmental Monitoring Centre Station，Dongguan 523000，China*

[4] *University of Chinese Academy of Sciences, Beijing 100049, China*

Corresponding author. Email address: cpan@gig.ac.cn

Table S1

Measured yields (mg/g TOC) of gas components

Time (h)	EASY %Ro	S (mg)	C_1	C_2H_6	C_2H_4 ×10^{-3}	C_3H_8	C_3H_6 ×10^{-3}	*i*C_4	*n*C_4	*i*C_5	*n*C_5	ΣC_{1-5}	$C_1/\Sigma C_{1-5}$ (wt)	H_2 ×10^{-3}	CO_2	H_2S
whole rock Lucaogou																
0	0.96	127.11	4.46	3.30	6.62	3.53	33.50	0.78	2.17	0.78	1.52	16.59	0.269	24.98	90.28	1.91
0	0.96	137.8	4.89	3.70	7.22	4.07	36.62	0.90	2.55	0.90	1.75	18.80	0.260	22.12	99.35	1.88
24	1.19	117.67	12.41	10.80	9.76	12.43	55.62	2.25	8.68	2.49	5.98	55.11	0.225	52.62	130.88	3.90
24	1.19	120.43	12.66	11.09	8.26	12.76	56.31	2.33	9.00	2.65	6.42	56.98	0.222	51.67	136.89	4.95
48	1.29	119.11	16.39	14.29	9.64	16.27	52.96	2.83	11.54	3.32	8.38	73.08	0.224	60.26	137.67	4.88
48	1.29	113.19	16.24	14.09	10.14	16.08	52.25	2.78	11.25	3.23	8.10	71.85	0.226	83.14	140.22	3.98
96	1.42	93.52	20.75	18.16	11.46	21.41	53.97	3.60	15.04	4.08	10.05	93.16	0.223	44.43	154.70	1.50
96	1.42	88.04	20.84	18.61	12.17	22.26	58.22	3.75	15.62	4.16	10.06	95.38	0.218	81.96	154.82	1.60
144	1.49	130.46	26.58	23.78	8.80	28.11	53.80	4.90	19.10	4.43	10.10	117.07	0.227	47.94	149.30	2.26
192	1.55	163.39	26.86	24.20	7.49	28.94	64.67	4.65	18.99	4.14	9.37	117.23	0.229	46.15	126.22	2.32
240	1.60	176.37	25.71	23.12	7.81	27.36	44.26	4.38	17.72	3.76	8.60	110.71	0.232	53.79	123.65	2.79
288	1.64	180.69	33.30	30.10	7.62	36.29	47.57	5.83	23.65	5.09	11.32	145.65	0.229	51.66	129.01	2.34
336	1.68	151.51	37.00	31.88	10.10	37.46	51.01	6.44	26.35	6.81	16.38	162.38	0.228	63.71	143.35	3.73
384	1.71	173.27	42.15	38.11	8.83	46.02	50.97	7.60	29.85	6.03	13.34	183.15	0.230	60.15	134.25	3.26
432	1.73	160.7	42.01	37.78	10.00	45.45	47.11	7.50	29.61	6.30	14.28	182.98	0.230	57.57	134.45	2.43
480	1.76	146.33	43.64	39.80	11.51	49.06	53.35	8.25	32.54	6.77	15.24	195.37	0.223	83.27	133.81	2.64
528	1.78	103.74	60.70	50.82	18.44	58.87	51.69	10.16	39.12	10.22	23.79	253.76	0.239	89.24	179.52	1.77
576	1.80	150.62											0.228			
624	1.82	149.86											0.226			
672	1.84	110.61	64.32	54.53	17.30	64.20	59.17	10.70	39.95	8.15	15.94	257.87	0.249	86.98	185.82	1.90
whole rock Lucaogou plus water (water/ TOC=2)																

0	0.96	92.29	4.62	3.30	21.56	3.10	98.26	0.64	1.55	0.45	0.82	14.60	0.316	124.31	466.62	7.02
0	0.96	96.59	3.53	2.32	16.64	1.99	53.88	0.41	0.84	0.22	0.35	9.73	0.363	102.31	393.30	4.26
24	1.19	99.42	10.81	8.97	26.17	9.89	221.30	1.86	6.13	1.78	3.70	43.38	0.249	186.50	701.43	9.56
24	1.19	95.11	8.39	6.71	23.34	7.09	139.29	1.38	4.31	1.27	2.69	32.00	0.262	129.86	608.86	6.51
48	1.29	94.56	13.81	11.36	23.47	12.28	212.66	2.06	6.96	1.85	3.87	52.42	0.263	183.97	800.53	13.41
48	1.29	98.34	10.56	8.47	18.68	8.76	140.33	1.52	4.82	1.27	2.65	38.22	0.276	168.17	724.98	9.82
96	1.42	42.36	20.87	19.74	19.87	22.44	169.41	3.86	15.42	4.30	9.72	96.54	0.216	182.96	673.05	8.53
96	1.42	48.02	16.89	15.26	20.72	16.33	210.20	2.88	10.28	2.95	6.09	70.92	0.238	169.08	884.13	7.27
144	1.49	189.67	18.49	15.39	14.53	16.71	156.33	2.63	8.43	2.16	3.89	67.86	0.272	158.25	860.01	12.67
192	1.55	159.14	23.46	20.70	19.72	24.80	188.80	4.08	14.01	3.64	6.73	97.62	0.240	198.20	928.82	12.29
240	1.60	162.13	22.80	19.50	21.24	22.44	156.13	3.56	12.03	2.97	5.67	89.14	0.256	192.86	916.49	12.75
288	1.64	154.98	29.42	26.57	26.67	33.97	202.00	6.11	20.18	5.85	9.34	131.67	0.223	229.63	969.44	17.90
336	1.68	158.86	29.92	27.12	24.09	33.38	175.23	5.49	19.25	5.00	9.09	129.45	0.231	212.73	951.68	12.12
384	1.71	156.88	37.20	34.73	25.37	42.46	171.91	6.81	23.61	5.67	9.95	160.61	0.232	204.54	953.66	11.90
432	1.73	159.76	37.69	34.85	25.39	42.60	150.55	6.85	23.45	5.88	10.10	161.59	0.233	187.71	958.70	11.96
480	1.76	163.23	39.64	37.69	24.85	47.17	164.26	7.75	26.55	6.45	10.92	176.35	0.225	213.43	932.73	11.56
528	1.78	157.17	37.50	35.67	24.83	47.02	151.52	8.18	28.63	7.59	12.72	177.48	0.211	194.61	926.09	10.12
576	1.80	159.68	37.63	35.16	24.92	46.71	153.09	8.55	28.49	7.87	12.74	177.32	0.212	199.24	911.55	10.52
624	1.82	154.83	38.55	35.71	26.69	44.50	138.53	7.30	25.10	6.34	11.03	168.70	0.229	186.74	916.85	9.48
672	1.84	156.88	40.08	36.64	25.86	43.42	140.75	6.70	21.17	4.29	6.19	158.67	0.253	178.67	899.33	9.11
whole rock Lucaogou plus water (water/ TOC=4)																
0	0.96	93.81	3.78	2.51	26.92	2.29	87.42	0.50	1.08	0.33	0.55	11.15	0.339	118.02	603.74	8.34
0	0.96	99.19	3.00	2.00	24.69	1.84	83.48	0.42	0.86	0.26	0.40	8.89	0.338	109.95	583.92	6.05
24	1.19	92.63	8.03	6.31	23.13	6.70	154.09	1.39	3.91	1.15	2.13	29.79	0.269	180.18	831.90	10.53
24	1.19	96.52	7.75	6.14	22.20	6.64	149.51	1.39	4.00	1.18	2.19	29.45	0.263	180.63	789.03	11.11
48	1.29	96.21	10.11	8.19	19.09	8.93	151.63	1.71	5.21	1.48	2.83	38.64	0.262	184.59	866.91	11.65

48	1.29	98.53	9.93	8.02	20.97	8.64	144.06	1.64	4.94	1.37	2.58	37.29	0.266	180.82	840.19	10.60
96	1.42	43.37	16.02	13.31	24.71	16.14	210.92	3.15	10.84	3.62	6.90	70.21	0.228	235.48	987.65	11.57
96	1.42	43.86	14.98	12.43	24.43	14.50	195.98	2.68	8.85	2.64	4.92	61.20	0.245	249.24	963.93	11.13
144	1.49	157.15	17.50	15.10	20.94	18.84	185.67	3.58	11.73	3.41	6.15	76.51	0.229	189.98	934.85	9.63
192	1.55	139.32	23.53	20.67	25.27	25.89	207.73	4.58	14.92	3.98	6.61	100.41	0.234	206.03	952.28	12.43
240	1.60	141.04	22.82	19.81	24.96	23.91	205.20	4.19	14.08	3.95	7.23	96.22	0.237	186.19	945.68	13.30
288	1.64	136.62	29.35	26.06	26.89	31.94	195.67	5.39	17.80	4.82	7.96	123.53	0.238	205.11	965.03	13.77
336	1.68	140.47	30.09	27.22	28.88	34.45	203.22	6.09	20.76	5.91	10.14	134.89	0.223	196.91	950.01	12.48
384	1.71	137.14	36.85	34.74	29.02	44.22	208.16	7.69	26.55	7.29	12.19	169.76	0.217	210.55	938.57	13.68
432	1.73	136.09	36.32	34.08	28.68	46.41	172.10	8.75	29.38	8.62	13.13	176.89	0.205	217.42	963.38	14.14
480	1.76	138.57	40.58	38.31	30.38	49.09	194.06	8.67	29.73	8.19	13.46	188.24	0.216	214.95	939.17	14.07
528	1.78	132.95	36.24	33.92	28.21	45.45	167.86	8.32	28.56	8.21	13.24	174.13	0.208	214.70	913.48	13.28
576	1.80	140.01	37.30	34.66	25.14	47.48	160.52	8.91	29.33	8.23	12.11	178.22	0.209	198.33	911.54	12.83
624	1.82	132.56	38.61	35.97	25.98	48.82	151.70	9.20	30.85	9.17	14.10	186.90	0.207	199.49	922.07	12.61
672	1.84	136.42	39.61	36.63	23.00	47.69	150.87	8.74	28.24	7.46	10.16	178.71	0.222	188.58	895.61	11.50
Kerogen Saergan																
0	1.16	86.43	4.65	2.08	1.95	0.61	2.81	0.08	0.10	0.01	0.02	7.57	0.615	9.90	18.02	4.02
0	1.16	91.47	4.74	2.21	2.39	0.63	2.47	0.09	0.11	0.01	0.03	7.82	0.607	9.21	12.39	2.32
24	1.28	91.43	11.33	6.83	2.76	3.23	2.09	0.48	0.73	0.08	0.16	22.83	0.496	18.51	15.22	6.01
24	1.28	85.33	11.71	7.09	2.76	3.35	2.24	0.49	0.77	0.09	0.17	23.67	0.495	15.05	15.60	5.85
48	1.35	87.11	14.08	8.25	3.48	4.02	2.39	0.60	0.88	0.11	0.18	28.12	0.501	20.48	15.97	4.74
48	1.35	91.87	13.74	7.84	3.67	3.74	2.83	0.56	0.82	0.10	0.17	26.97	0.509	17.97	15.50	4.88
72	1.40	81.93	17.74	9.87	3.91	5.09	2.54	0.76	1.17	0.15	0.25	35.05	0.506	21.14	15.59	4.89
72	1.40	82.32	17.51	9.60	4.30	4.86	2.32	0.72	1.09	0.14	0.23	34.16	0.513	19.75	15.32	4.49
96	1.45	83.86	18.92	10.42	4.82	5.36	2.28	0.79	1.22	0.16	0.26	37.13	0.509	20.02	16.00	5.16
96	1.45	85.58	18.94	10.45	4.72	5.37	2.43	0.80	1.23	0.16	0.26	37.21	0.509	20.65	16.07	4.76

144	1.52	151.58	18.84	12.67	2.00	7.54	2.06	1.01	1.84	0.27	0.33	42.50	0.443	14.15	23.96	11.37
192	1.57	161.85	19.45	12.94	1.77	7.80	1.93	1.03	1.88	0.27	0.32	43.69	0.445	15.11	23.18	11.83
240	1.62	151.99	18.62	12.58	2.11	7.42	2.17	0.98	1.71	0.24	0.28	41.85	0.445	12.18	23.25	10.08
288	1.65	151.9	24.53	14.61	2.22	7.81	2.28	0.96	1.59	0.23	0.25	49.98	0.491	16.49	24.53	9.15
336	1.69	142.48	26.07	15.05	2.60	7.89	2.80	0.96	1.54	0.22	0.23	51.94	0.502	16.22	24.88	9.22
384	1.72	148.95	30.33	16.25	2.26	7.80	1.86	0.89	1.32	0.18	0.17	56.95	0.533	15.56	25.46	8.25
432	1.74	142.99	31.74	17.40	2.47	8.45	2.18	0.95	1.32	0.19	0.17	60.22	0.527	18.50	25.97	8.62
480	1.77	153.21	32.40	16.84	2.42	7.66	2.04	0.83	1.13	0.15	0.12	59.14	0.548	11.49	25.49	7.65
528	1.79	148.73	30.09	16.25	2.49	7.82	1.75	0.84	1.22	0.15	0.13	56.50	0.533	30.18	24.23	7.68
576	1.81	147.73	31.40	16.76	2.62	7.87	2.00	0.88	1.15	0.15	0.12	58.34	0.538	40.17	24.72	6.43
624	1.83	145.54	38.13	18.32	2.78	7.61	2.38	0.77	0.92	0.11	0.08	65.96	0.578	44.66	26.49	7.93
672	1.85	144.47	32.22	16.49	2.91	7.71	2.76	0.83	0.98	0.11	0.07	58.43	0.552	36.48	24.08	7.27
Kerogen Saergan plus water (water/ TOC=2)																
0	1.16	82.05	4.71	2.24	2.26	0.65	2.54	0.09	0.10	0.01	0.02	7.82	0.603	18.12	17.92	7.93
0	1.16	83.76	4.10	2.13	2.81	0.63	2.49	0.10	0.08	0.01	0.01	7.06	0.581	6.95	18.69	6.73
24	1.28	83.47	12.17	7.82	3.23	3.86	2.91	0.54	0.86	0.10	0.17	25.53	0.476	20.68	17.61	8.67
24	1.28	80.28	10.97	7.58	3.36	3.82	3.03	0.50	0.91	0.10	0.19	24.08	0.456	14.95	18.33	9.59
48	1.35	84.08	14.86	9.82	3.81	4.85	3.30	0.66	0.99	0.16	0.16	31.52	0.472	29.11	18.81	13.01
48	1.35	83.12	11.38	7.14	3.85	3.00	3.34	0.42	0.50	0.09	0.07	22.61	0.504	13.61	19.46	10.27
72	1.40	71.05	19.33	11.60	4.74	6.05	3.91	1.18	1.18	0.33	0.18	39.86	0.485	44.14	21.19	19.80
72	1.40	81.99	16.34	10.15	4.31	4.94	3.39	0.85	0.89	0.21	0.12	33.51	0.488	21.89	20.79	16.26
96	1.45	81.3	20.46	11.90	4.56	5.99	3.41	1.09	1.13	0.32	0.17	41.06	0.498	34.36	20.81	16.26
96	1.45	81.01	16.89	9.95	4.78	5.12	3.43	0.74	1.21	0.15	0.26	34.33	0.492	18.70	17.58	7.10
144	1.52	119.06	16.08	9.55	3.25	6.15	3.50	2.34	2.11	1.49	0.70	38.44	0.418	54.71	34.51	23.01
192	1.57	116.91	16.05	9.47	3.02	6.10	3.71	2.53	2.26	1.92	0.91	39.24	0.409	46.43	34.78	26.26
240	1.62	116.05	16.00	9.50	3.05	6.34	3.74	2.51	2.21	1.67	0.77	39.01	0.410	45.65	34.96	22.56

288	1.65	125.64	20.48	11.59	3.35	6.94	2.90	2.55	1.87	1.46	0.59	45.49	0.450	44.77	35.66	26.22
336	1.69	117.09	23.42	12.30	3.31	7.83	3.70	3.03	2.57	1.84	0.81	51.80	0.452	50.29	36.02	23.95
384	1.72	124.64	27.29	13.16	3.38	8.39	2.78	3.18	2.74	1.86	0.84	57.46	0.475	51.29	36.76	20.81
432	1.74	116.51	27.62	13.47	3.61	8.73	3.43	3.40	2.85	1.86	0.93	58.86	0.469	44.07	36.91	19.01
480	1.77	119.56	28.41	13.92	3.52	7.92	1.89	2.79	1.86	1.11	0.41	56.42	0.504	41.50	37.84	24.77
528	1.79	123.85	27.29	13.27	3.67	8.25	2.38	3.06	2.32	1.46	0.58	56.24	0.485	49.04	33.99	14.24
576	1.81	131.03	27.18	13.30	3.73	9.19	3.05	3.87	2.98	2.22	0.95	59.71	0.455	59.54	34.72	19.92
624	1.83	122.11	36.37	16.10	3.72	8.04	3.27	1.93	1.14	0.33	0.11	64.04	0.568	73.69	40.76	23.55
672	1.85	114.86	28.94	13.92	3.81	7.61	2.72	2.39	1.29	0.51	0.13	54.80	0.528	60.83	37.04	20.32
Kerogen Saergan plus water (water/ TOC=4)																
0	1.16	62.92	4.45	2.21	3.75	0.67	2.76	0.14	0.08	0.02	0.01	7.59	0.587	21.96	23.47	11.64
0	1.16	63.36	4.06	1.77	3.46	0.53	3.01	0.10	0.07	0.01	0.01	6.56	0.619	17.81	24.60	5.76
24	1.28	62.21	11.80	7.36	3.79	2.92	4.18	0.45	0.43	0.08	0.05	23.10	0.511	32.61	23.96	16.41
48	1.35	63.77	12.10	7.75	3.96	3.41	3.81	0.55	0.54	0.11	0.06	24.53	0.493	21.03	20.98	12.96
48	1.35	62.20	13.37	8.52	4.33	3.75	4.18	0.52	0.65	0.10	0.09	27.01	0.495	21.81	19.90	11.05
72	1.40	65.51	17.60	11.42	4.37	5.91	3.97	1.24	1.07	0.30	0.15	37.69	0.467	32.57	24.01	21.30
72	1.40	61.48	18.43	11.77	4.66	6.20	4.23	1.33	1.12	0.32	0.15	39.32	0.469	38.83	23.93	20.97
96	1.45	64.64	17.53	11.11	4.95	5.98	4.56	1.49	1.07	0.35	0.14	37.68	0.465	46.42	24.37	22.88
96	1.45	60.54	18.55	11.70	5.29	6.21	5.16	1.52	1.10	0.37	0.15	39.60	0.468	46.35	25.22	22.60
144	1.52	108.51	14.71	8.61	3.57	6.26	4.96	2.71	2.26	1.70	0.76	37.01	0.397	84.43	34.96	28.55
192	1.57	106.68	14.40	8.52	4.10	6.33	5.04	2.76	2.31	1.85	0.84	37.03	0.389	74.23	36.58	35.34
240	1.62	105.30	13.93	8.16	4.00	6.20	4.78	2.74	2.42	1.98	0.94	36.38	0.383	74.94	36.19	29.48
288	1.65	104.26	18.30	10.11	3.88	7.08	3.99	3.29	2.26	1.96	0.72	43.74	0.418	76.36	38.29	38.12
336	1.69	105.87	20.20	10.70	3.66	7.58	3.77	3.31	2.57	2.02	0.84	47.24	0.428	72.53	37.64	35.03
384	1.72	111.05	24.25	12.10	3.64	7.72	3.44	3.16	2.08	1.52	0.56	51.40	0.472	67.24	38.16	32.19
432	1.74	109.98	23.89	11.76	3.22	7.76	3.31	3.12	2.28	1.58	0.63	51.02	0.468	67.97	40.21	40.28

480	1.77	101.94	26.87	13.07	4.13	8.50	2.89	3.36	2.16	1.35	0.46	55.78	0.482	64.62	38.36	29.84
528	1.79	106.15	24.84	11.94	3.81	8.33	3.76	3.52	2.61	1.86	0.74	53.87	0.461	77.06	35.11	21.41
576	1.81	106.8	24.85	12.47	3.15	7.91	5.04	3.30	1.87	1.29	0.40	52.10	0.477	81.36	38.16	31.66
624	1.83	105.13	28.65	12.55	2.88	8.20	4.13	3.28	2.51	1.51	0.69	57.39	0.499	108.78	38.36	30.23
672	1.85	100.43	26.95	12.66	2.68	8.13	4.32	3.46	2.07	1.31	0.40	54.98	0.490	81.70	40.38	32.84
Kerogen Wuerhe																
0	1.21	90.19	3.82	3.96	2.84	3.72	9.84	0.67	2.58	0.52	1.98	17.27	0.221	19.82	18.53	6.85
0	1.21	90.15	4.13	4.21	3.14	3.99	10.15	0.77	2.90	0.65	2.36	19.02	0.217	23.46	18.06	6.46
48	1.37	87.92	12.56	11.62	7.66	11.56	12.78	1.58	8.33	1.50	5.74	52.92	0.237	38.27	21.28	6.91
96	1.47	87.17	13.73	12.54	--	12.59	11.30	1.67	8.70	1.44	5.47	56.14	0.245	32.89	21.72	6.92
96	1.47	87.51	15.87	14.02	--	14.42	13.95	1.92	10.45	1.86	6.98	65.53	0.242	37.93	22.49	7.05
144	1.53	86.59	16.65	14.88	--	15.36	12.33	2.00	10.51	1.72	6.32	67.46	0.247	37.86	22.76	7.14
192	1.58	85.90	24.81	21.74	--	23.64	12.82	3.01	14.53	2.26	6.90	96.91	0.256	52.79	27.62	7.41
192	1.58	78.62	26.76	23.37	--	25.34	14.47	3.22	16.02	2.41	7.74	104.87	0.255	56.64	28.85	8.22
240	1.63	84.41	27.10	23.50	9.89	26.24	16.76	3.51	17.80	2.87	9.29	110.35	0.246	48.81	27.09	6.59
288	1.66	83.98	24.68	21.43	9.46	22.75	13.21	2.96	15.40	2.59	8.82	98.65	0.250	51.16	25.59	7.24
288	1.66	84.27	24.47	21.07	--	22.17	13.00	2.87	14.92	2.51	8.47	96.49	0.254	46.19	26.51	6.77
336	1.69	83.73	25.69	22.17	9.65	23.69	13.09	3.12	16.27	2.83	9.45	103.24	0.249	45.74	25.48	6.55
384	1.72	82.47	30.09	25.67	14.36	27.33	13.47	3.54	17.77	2.89	9.24	116.56	0.258	53.96	28.64	6.13
384	1.72	80.88	29.43	25.13	12.82	27.07	13.38	3.53	17.61	2.88	9.22	114.91	0.256	51.73	28.03	6.45
480	1.77	81.06	31.90	26.94	13.29	29.59	13.18	4.00	19.72	3.31	10.39	125.87	0.253	55.07	27.52	6.78
576	1.82	78.59	37.87	32.34	16.10	35.98	14.30	4.94	23.50	4.06	12.19	150.91	0.251	63.99	31.13	6.69
576	1.82	79.19	37.86	32.33	16.32	36.10	16.46	4.92	23.51	3.91	11.68	150.34	0.252	62.10	29.50	6.67
672	1.85	79.60	42.58	36.04	--	39.53	16.86	5.44	26.80	4.80	14.85	170.06	0.250	71.63	34.87	7.93
Kerogen Wuerhe plus water (water/ TOC=2)																
0	1.21	89.31	4.17	3.88	8.29	3.03	37.12	0.65	1.35	0.43	0.66	14.21	0.293	59.27	26.45	13.80

0	1.21	90.43	3.86	3.23	7.94	2.26	29.61	0.55	0.87	0.29	0.33	11.41	0.338	54.35	29.62	10.10
48	1.37	88.80	13.19	12.30	8.49	11.81	37.96	2.66	5.89	2.36	2.63	50.88	0.259	106.09	31.50	28.04
96	1.47	87.66	14.99	13.64	7.99	13.63	28.32	3.16	7.26	2.72	2.95	58.38	0.257	95.13	31.66	23.62
96	1.47	88.00	16.91	15.28	9.03	16.62	34.36	4.38	9.90	4.48	4.59	72.20	0.234	108.23	33.33	29.74
144	1.53	86.36	21.42	18.61	13.56	20.48	35.98	5.25	11.49	4.81	4.79	86.90	0.246	117.16	36.79	31.37
192	1.58	86.34	24.33	20.79	14.66	23.92	50.60	6.55	14.07	5.97	5.70	101.40	0.240	122.29	37.35	29.54
192	1.58	86.19	22.99	19.85	14.84	22.07	35.56	5.45	11.36	4.18	4.01	89.96	0.256	116.94	36.60	29.59
240	1.63	83.44	26.77	22.45	--	26.26	33.25	7.77	15.75	7.70	6.71	113.43	0.236	130.41	38.84	34.88
288	1.66	84.07	24.25	20.47	12.81	22.82	28.71	6.06	13.30	6.03	5.84	98.82	0.245	101.92	36.23	26.29
288	1.66	84.34	26.38	22.14	15.64	25.43	29.60	6.77	14.06	5.43	5.04	105.30	0.251	116.24	40.51	26.46
336	1.69	83.71	32.37	26.64	19.46	32.03	30.49	8.88	19.41	8.76	8.00	136.16	0.238	134.84	42.55	32.65
384	1.72	81.93	30.86	25.36	17.42	28.91	51.63	7.39	14.27	5.44	4.86	117.16	0.263	123.07	40.33	30.91
384	1.72	82.16	32.52	26.58	17.70	31.32	29.54	8.91	19.12	8.71	7.93	135.13	0.241	128.20	41.94	31.62
480	1.77	81.56	34.33	28.16	19.81	33.56	28.40	9.58	20.81	8.39	7.30	142.18	0.241	122.41	42.75	33.56
576	1.82	79.13	37.07	29.70	--	35.37	22.44	10.66	21.70	10.22	9.05	153.79	0.241	118.28	43.75	31.57
576	1.82	79.61	37.49	30.23	--	36.12	24.04	10.94	21.46	9.72	7.97	153.95	0.244	122.91	43.33	31.48
672	1.85	78.55	42.29	33.91	--	41.69	24.16	12.59	23.26	9.02	7.14	169.92	0.249	128.23	48.70	33.87
Kerogen Fengcheng																
0	1.07	72.91	14.28	6.10	6.36	7.58	29.77	3.70	3.24	2.72	1.83	39.49	0.362	27.02	32.75	27.62
48	1.32	70.18	35.26	18.97	10.51	21.27	20.41	10.48	9.97	8.62	5.10	109.70	0.321	50.14	33.71	26.50
48	1.32	66.74	40.43	21.87	12.63	24.67	23.09	11.87	11.35	9.36	5.48	125.06	0.323	60.61	34.60	26.96
96	1.43	69.27	47.48	25.55	17.33	29.23	20.37	13.95	13.76	10.90	6.34	147.26	0.322	65.47	35.16	26.94
144	1.50	68.80	53.18	28.40	19.90	32.75	20.51	15.61	15.87	12.91	7.61	166.37	0.320	77.35	35.07	26.87
144	1.50	71.55	49.05	26.13	15.02	29.58	16.99	14.14	14.10	11.78	7.00	151.81	0.323	68.36	35.42	27.14
240	1.61	69.38	50.03	27.20	16.45	34.47	18.39	16.37	16.36	11.06	6.29	161.82	0.309	54.04	34.90	28.26
240	1.61	65.81	50.80	26.89	15.05	33.04	13.85	15.97	16.25	12.86	7.39	163.22	0.311	55.50	36.95	28.55

288	1.65	64.47	47.25	24.68	12.42	29.59	11.11	14.17	14.17	10.87	6.08	146.83	0.322	47.59	35.40	27.35
336	1.68	67.66	51.57	26.70	0.00	32.74	12.83	15.71	15.94	12.16	6.85	161.68	0.319	55.77	36.43	28.15
336	1.68	64.58	54.04	28.27	15.33	34.89	13.78	17.19	17.71	13.79	7.95	173.87	0.311	68.19	38.04	29.40
384	1.71	66.12	63.05	33.45	19.44	41.02	18.06	19.28	20.51	14.99	9.04	201.38	0.313	75.80	39.33	30.39
480	1.76	63.53	60.66	31.93	16.25	39.30	13.67	18.51	19.36	13.86	8.12	191.76	0.316	66.92	40.16	31.03
480	1.76	61.30	66.02	35.04	18.90	43.32	15.58	20.49	22.22	16.52	10.38	214.02	0.308	70.86	40.94	31.63
576	1.81	62.57	64.39	33.98	16.83	41.40	13.18	19.31	20.61	15.18	9.27	204.17	0.315	70.17	40.82	31.55
672	1.85	61.55	72.00	37.98	19.03	46.57	14.22	22.02	23.89	18.17	11.40	232.06	0.310	75.06	44.88	31.82
672	1.85	61.27	73.08	39.11	23.38	47.79	15.94	21.89	23.79	16.24	10.41	232.34	0.315	80.11	44.92	34.71
Kerogen Fengcheng plus water (water/ TOC=2)																
0	1.07	71.38	14.29	4.86	12.99	4.92	51.09	2.15	1.36	1.04	0.59	29.28	0.488	58.06	37.95	35.33
48	1.32	70.13	30.20	15.56	11.41	20.09	57.26	9.77	8.98	7.13	3.97	95.77	0.315	95.56	51.20	39.56
48	1.32	71.49	30.76	15.85	12.08	20.19	61.03	9.61	8.69	6.67	3.65	95.49	0.322	88.40	53.69	41.49
96	1.43	68.12	35.93	18.78	12.99	23.97	43.02	10.71	9.72	6.61	3.51	109.29	0.329	101.44	55.91	43.20
144	1.50	69.37	40.62	21.34	17.92	27.69	39.11	12.56	12.03	8.28	4.47	127.05	0.320	103.60	59.37	45.87
144	1.50	67.85	38.99	20.59	17.48	27.56	39.07	12.54	11.84	8.06	4.33	123.96	0.315	103.52	55.52	42.90
192	1.56	69.33	49.23	26.33	22.49	35.34	48.53	16.19	16.61	11.59	6.42	161.78	0.304	120.67	69.63	53.80
240	1.61	66.61	55.91	29.67	23.72	40.19	46.93	18.50	19.84	14.18	8.11	186.47	0.300	138.13	73.59	56.86
240	1.61	66.10	--	--	--	--	--	--	--	--	--	--	--	--	--	--
288	1.65	65.92	56.52	29.53	0.00	39.59	34.16	19.12	20.46	15.67	8.86	189.79	0.298	122.18	75.15	58.07
336	1.68	65.73	59.88	31.86	22.43	43.45	34.67	20.06	22.79	16.87	10.05	205.02	0.292	138.56	75.25	58.15
336	1.68	68.27	61.83	32.97	22.84	44.33	41.97	20.08	22.41	16.10	9.51	207.28	0.298	130.94	80.05	61.86
384	1.71	64.68	65.65	35.05	25.40	47.38	43.63	21.02	23.67	15.57	9.14	217.54	0.302	152.65	82.78	63.96
480	1.76	65.99	70.59	37.86	28.09	51.36	37.83	22.66	25.98	16.23	9.63	234.38	0.301	146.96	84.13	65.01
480	1.76	64.14	70.97	38.34	24.30	51.87	34.18	23.19	27.20	18.97	11.71	242.29	0.293	144.71	84.78	65.51
576	1.81	60.05	75.53	40.59	28.06	54.97	35.42	23.26	26.23	15.10	9.00	244.74	0.309	164.17	89.43	69.10

672	1.85	64.27	77.64	41.85	30.81	56.74	33.10	24.83	29.44	18.95	11.78	261.30	0.297	147.71	95.00	73.41
672	1.85	62.47	77.52	41.55	27.68	56.32	32.88	24.64	28.55	18.74	11.07	258.45	0.300	146.79	93.41	72.20

Time: heating time being hold for 372 °C; S: the amount of samples (whole rock Lucaogou or kerogens Saergan, Wuerhe and Fengcheng)

Table S2

Measured carbon isotopes ($\delta^{13}C$ ‰ PDB) of gas components

Time (h)	EASY %Ro	$\delta^{13}C_1$				$\delta^{13}C_2$				$\delta^{13}C_3$				$\delta^{13}C_{CO2}$			
		1	2	3	av	1	2	3	av	1	2	3	av	1	2	3	av
whole rock Lucaogou																	
0	0.96	-46.76	-46.52		-46.64	-36.06	-36.02		-36.04	-34.64	-34.74		-34.69	7.25	7.37		7.31
0	0.96	-46.84	-47.00		-46.92	-36.26	-36.09		-36.17	-34.26	-34.41		-34.33	7.59	7.68		7.64
24	1.19	-46.71	-46.65		-46.68	-35.33	-35.12		-35.23	-34.31	-34.26		-34.28	7.88	7.91		7.90
24	1.19	-46.39	-46.54	-46.46	-46.46	-35.49	-35.79	-35.61	-35.63	-34.22	-34.01	-34.12	-34.12	7.86	7.86		7.86
48	1.29	-45.33	-45.16		-45.25	-35.31	-35.08		-35.20	-34.49	-34.43		-34.46	7.72	7.51		7.61
48	1.29	-45.48	-45.66	-45.58	-45.57	-35.36	-35.42	-35.58	-35.45	-34.60	-34.40	-34.17	-34.39	9.53	9.94		9.73
96	1.42	-45.62	-45.30		-45.46	-35.28	-34.91		-35.09	-34.15	-33.93		-34.04	7.17	7.23		7.20
96	1.42	-45.02	-44.82	-44.98	-44.94	-34.98	-34.78	-34.82	-34.82	-34.26	-34.01	-33.88	-34.05	6.30	6.66		6.48
144	1.49	-44.98	-44.68		-44.83	-35.12	-34.90		-34.96	-34.42	-34.26		-34.34	6.40	6.04		6.22
192	1.55	-44.92	-44.75	-44.73	-44.80	-35.10	-34.86	-34.72	-34.89	-34.38	-34.18	-34.11	-34.22	5.98	5.77		5.88
240	1.60	-44.92	-44.86		-44.89	-35.47	-35.37		-35.42	-34.12	-33.98		-34.05	6.49	6.21		6.35
288	1.64	-44.87	-44.59	-44.46	-44.64	-35.14	-34.99	-34.90	-35.01	-34.12	-33.89	-33.93	-33.98	5.72	5.40		5.56
336	1.68	-43.75	-43.59		-43.67	-35.72	-35.56		-35.64	-34.56	-34.44		-34.50	7.78	7.66		7.72
384	1.71	-44.74	-44.56		-44.65	-35.97	-35.78		-35.88	-34.42	-34.06		-34.24	6.02	5.64		5.83

432	1.73	-44.22	-43.85	-44.02	-44.03	-35.81	-35.61	-35.68	-35.70	-34.21	-33.82	-33.79	-33.94	6.10	5.54	5.82
480	1.76	-44.02	-43.98		-44.00	-35.98	-35.86		-35.92	-34.15	-34.05		-34.10	5.68	5.38	5.53
528	1.78	-44.51	-44.63		-44.57	-36.14	-36.00		-36.07	-34.44	-34.38		-34.41	4.86	4.62	4.74
576	1.80	-44.64	-44.48		-44.56	-36.29	-36.07		-36.18	-34.74	-34.68		-34.71	6.26	6..16	6.21
624	1.82	-44.03	-43.97		-44.00	-36.10	-35.82		-35.96	-34.66	-34.52		-34.59	5.23	4.97	5.10
672	1.84	-43.25	-43.33		-43.29	-35.68	-35.56		-35.62	-34.38	-34.48		-34.43	6.38	6.66	6.52
whole rock Lucaogou plus water (water/ TOC=2)																
0	0.96	-46.57	-46.63		-46.60	-36.40	-36.22		-36.31	-35.94	-35.78		-35.86	13.50	13.52	-13.51
0	0.96	-46.44	-46.36		-46.40	-36.36	-36.20		-36.26	-35.42	-35.28		-35.35	13.62	14.00	-13.81
24	1.19	-45.85	-45.73		-45.79	-35.60	-35.30		-35.45	-35.13	-35.01		-35.07	13.39	13.10	-13.25
24	1.19	-46.03	-45.77		-45.90	-35.48	-35.28		-35.38	-35.09	-34.91		-35.00	14.19	14.29	14.25
48	1.29	-45.35	-45.25		-45.30	-35.77	-35.65		-35.71	-35.32	-35.12		-35.22	13.73	13.87	13.80
48	1.29	-45.86	-45.64		-45.75	-35.54	-35.48		-35.51	-35.27	-35.15		-35.21	14.28	14.21	14.25
96	1.42	-45.07	-44.87		-44.97	-34.56	-34.46		-34.51	-34.38	-34.28		-34.31	12.26	12.34	12.30
96	1.42	-44.56	-44.44		-44.50	-34.58	-34.34		-34.46	-34.74	-34.56		-34.65	12.17	12.32	12.24
144	1.49	-44.59	-44.51		-44.55	-34.32	-34.16		-34.24	-34.51	-34.33		-34.42	12.41	12.48	12.48
192	1.55	-44.64	-44.38		-44.51	-35.35	-35.19		-35.27	-34.38	-34.20		-34.29	12.98	13.06	-13.02
240	1.60	-44.32	-44.18		-44.25	-35.43	-35.31		-35.37	-34.29	-34.19		-34.24	12.78	12.94	12.86
288	1.64	-43.94	-43.72		-43.83	-3621	-36.17		-36.19	-34.12	-34.02		-34.07	11.79	11.83	11.81
336	1.68	-43.97	-43.81		-43.89	-36.72	-36.57		-36.65	-34.50	-34.48		-34.49	12.29	12.53	12.41
384	1.71	-44.32	-44.16		-44.24	-36.93	-36.83		-36.88	-34.87	-34.81		-34.84	12.46	12.70	12.58
432	1.73	-44.21	-44.39		-44.30	-36.89	-36.83		-38.86	-34.78	-34.64		-34.71	12.39	12.65	12.52
480	1.76	-44.45	-44.27		-44.36	-36.92	-36.84		-36.88	-34.54	-34.42		-34.48	12.62	12.76	12.69
528	1.78	-45.04	-44.88		-44.96	-37.31	-37.21		-37.26	-34.95	-34.71		-34.83	12,76	12.88	12.82
576	1.80	-45.12	-45.08		-45.10	-37.56	-37.42		-37.49	-34.69	-34.61		-34.65	12.71	12.83	12.77
624	1.82	-45.42	-45.26		-45.34	-37.49	-37.71		-37.60	-35.01	-35.07		-35.04	12.62	12.30	12.46

672	1.84	-45.13	-44.99	-45.06	-37.49	-37.40	-37.44	-34.85	-34.71	-34.78	11.52	11.40	11.46
whole rock Lucaogou plus water (water/ TOC=4)													
0	0.96	-45.29	-45.21	-45.25	-36.18	-36.12	-36.15	-35.86	-35.76	-35.81	13.72	13.90	13.86
0	0.96	-44.92	-44.80	-44.86	-35.88	-35.78	-35.83	-35.84	-35.75	-35.79	13.96	14.08	14.02
24	1.19	-44.25	-44.20	-44.22	-36.10	-35.89	-35.99	-35.93	-36.00	-35.96	13.36	13.58	13.47
24	1.19	-44.86	-44.74	-44.80	-35.67	-35.57	--35.62	-35.92	-35.76	-35.84	13.13	13.31	13.22
48	1.29	-44.94	-44.92	-44.93	-35.83	-35.74	-35.78	-36.01	-35.89	-35.95	13.10	13.14	13.12
48	1.29	-44.61	-44.56	-44.58	-35.64	-35.52	-35.58	-35.89	-35.81	-35.85	13.24	13.12	13.18
96	1.42	-44.58	-44.36	-44.47	-35.88	-35.80	-35.84	-35.42	-35.34	-35.38	12.86	12.72	12.74
96	1.42	-44.92	-44.68	-44.80	-35.52	-35.40	-35.46	-35.31	-35.19	-35.25	12.76	12.82	12.79
144	1.49	-43.99	-43.87	-43.93	-35.60	-35.56	-35.58	-34.98	-34.74	-34.86	12.48	12.59	12.53
192	1.55	-44.45	-44.21	-44.33	-35.90	-35.76	-35.83	-34.54	-34.32	-34.43	12.60	12.72	12.66
240	1.60	-43.98	-44.10	-44.04	-35.54	-35.40	-35.47	-34.43	-34.30	-34.36	12.52	12.64	12.58
288	1.64	-44.04	-43.80	-43.92	-36.16	-36.12	-36.14	-34.42	-34.32	-34.37	11.74	12.06	11.90
336	1.68	-43.39	-43.33	-43.36	-37.01	-36.97	-36.99	-34.42	-34.40	-34.41	13.52	13.48	13.50
384	1.71	-44.53	-44.29	-44.41	-37.09	-37.03	-37.06	-34.65	-34.61	-34.63	12.45	12.65	12.55
432	1.73	-44.54	-44.42	-44.48	-37.29	-37.21	-37.25	-35.53	-35.41	-35.47	12.41	12.43	12.42
480	1.76	-44.35	-44.19	-44.27	-37.32	-37.08	-37.20	-34.91	-34.75	-34.83	12.14	12.01	12.07
528	1.78	-44.55	-44.44	-44.49	-37.72	-37.50	-37.61	-35.18	-35.12	-35.15	11.70	11.94	11.82
576	1.80	-44.64	-44.40	-44.52	-37.65	-37.47	-37.56	-35.40	-35.24	-35.32	11.40	11.75	11.57
624	1.82	-44.79	-44.59	-44.69	-37.52	-37.44	-37.48	-35.57	-35.50	-35.53	11.82	11.64	11.73
672	1.84	-44.82	-44.70	-44.76	-37.58	-37.38	-37.48	-35.43	-35.39	-35.41	12.02	11.77	11.89
Kerogen Saergan													
0	1.16	-43.56	-43.40	-43.48	-34.55	-34.46	-34.50	-31.94	-31.82	-31.88	-27.64	-27.53	-27.58
0	1.16	-43.44	-43.22	-43.33	-34.20	-34.04	-34.12	-31.12	-31.04	-31.08	-27.66	-27.62	-27.64
24	1.28	-42.04	-41.88	-41.96	-32.01	-31.97	-31.99	-30.29	-30.16	-30.23	-27.38	-27.30	-27.34

24	1.28	-42.26	-42.05	-42.15	-32.21	-32.09	-32.15	-30.32	-30.24	-30.28	-27.43	-27.33	-27.38
48	1.35	-42.35	-42.29	-42.32	-32.48	-32.40	-32.44	-30.14	-29.96	-30.05	-27.39	-27.35	-27.37
48	1.35	-42.24	-42.12	-42.18	-32.09	-32.01	-32.05	-30.78	-30.52	-30.65	-27.76	-27.52	-27.64
72	1.40	-41.63	-41.45	-41.54	-31.78	-31.67	-31.73	-30.16	-30.04	-30.10	-27.52	-27.44	-27.48
72	1.40	-41.86	-41.70	-41.78	-32.06	-31.94	-32.00	-30.11	-29.87	-29.99	-27.61	-27.49	-27.55
96	1.45	-40.88	-40.84	-40.86	-31.12	-30.98	-31.05	-30.11	-2999	-30.05	-27.66	-27.60	-27.63
96	1.45	-40.97	-40.85	-40.91	-31.01	-30.77	-30.89	-30.06	-29.98	-30.02	-27.60	-27.56	-27.58
144	1.52	-41.12	-41.04	-41.08	-31.04	-30.88	-30.96	-29.32	-29.20	-29.26	-26.84	-26.72	-26.78
192	1.57	-40.78	-40.60	-40.69	-31.09	-30.87	-30.98	-28.76	-28.64	-28.70	-26.99	-26.89	-26.94
240	1.62	-41.06	-40.84	-40.95	-30.76	-30.68	-30.72	-29.31	-29.11	-29.21	-27.64	-27.54	-27.59
288	1.65	-40.67	-40.43	-40.55	-29.74	-29.56	-29.65	-27.74	-27.68	-27.71	-27.49	-27.45	-27.47
336	1.69	-40.44	-40.40	-40.42	-29.92	-29.80	-29.86	-28.04	-27.88	-27.96	-27.06	-27.02	-27.04
384	1.72	-46.16	-40.06	-40.11	-29.14	-29.06	-29.10	-27.92	-27.76	-27.84	-27.12	-27.06	-27.09
432	1.74	-40.83	-40.76	-40.79	-30.23	-30.11	-30.17	-27.79	-27.65	-27.72	-27.74	-27.66	-27.70
480	1.77	-40.54	-40.48	-40.51	-30.08	-29.96	-30.02	-28.08	-27.88	-27.95	-28.18	-28.14	-28.16
528	1.79	-40.47	-40.35	-40.41	-30.33	-30.27	-30.30	-28.57	-28.45	-28.51	-28.37	-28.21	-28.29
576	1.81	-40.09	-39.97	-40.03	-29.97	-29.77	-29.87	-28.22	-28.10	-28.16	-27.96	-27.80	-27.88
624	1.83	-40.12	-40.11	-40.11	-29.68	-29.56	-29.62	-27.58	-27.40	-27.49	-28.18	-28.10	-28.14
672	1.85	-40.88	-40.76	-40.82	-30.11	-29.97	-30.04	-28.14	-28.08	-28.11	-28.22	-28.18	-28.20
Kerogen Saergan plus water (water/ TOC=2)													
0	1.16	-42.96	-42.90	-42.93	-34.01	-34.02	-34.01	-31.19	-31.03	-31.11	-29.70	-29.62	-29.66
0	1.16	-42.62	-42.50	-42.62	-34.25	-34.03	-34.14	-31.04	-30.82	-30.93	-29.07	-29.03	-29.05
24	1.28	-41.62	-41.58	-41.60	-32.07	-31.83	-31.95	-30.68	-30.50	-30.59	-28.66	-28.52	-28.59
24	1.28	-41.86	-41.74	-41.80	-32.14	-31.98	-32.06	-30.67	-30.57	-30.62	-28.86	-28.62	-28.74
48	1.35	-41.52	-41.38	-41.45	-31.98	-31.90	-31.94	-29.91	-29.85	-29.88	-28.74	-28.58	-28.66
48	1.35	-41.42	-41.26	-41.34	-32.12	-31.98	-32.05	-30.45	-30.21	-30.33	-28.91	-28.83	-28.87

72	1.40	-41.54	-41.26	-41.40	-31.93	-31.79	-31.86	-30.64	-30.46	-30.55	-28.48	-28.24	-28.36
72	1.40	-41.65	-41.57	-41.61	-32.21	-32.05	-32.13	-30.70	-3056	-30.63	-28.76	-28.56	-28.66
96	1.45	-41.40	-41.22	-41.31	-31.74	-31.62	-31.68	-30.08	-30.06	-30.07	-28.16	-27.94	-28.05
96	1.45	-41.84	-41.74	-41.79	-32.12	-32.06	-32.09	-30.70	-30.46	-30.58	-28.24	-28.12	-28.18
144	1.52	-41.56	-41.32	-41.44	-32.06	-31.94	-32.00	-30.22	-30.12	-30.17	-27.69	-27.57	-27.63
192	1.57	-41.06	-40.92	-40.99	-31.63	-31.39	-31.51	-30.18	-30.02	-30.10	-27.51	-27.31	-27.41
240	1.62	-40.47	-40.41	-40.44	-31.18	-31.16	-31.17	-29.62	-29.50	-29.56	-27.79	-27.65	-27.72
288	1.65	-40.49	-40.31	-40.40	-30.97	-30.83	-30.90	-29.41	-29.31	-29.36	-27.93	-27.85	-27.89
336	1.69	-40.28	-40.24	-40.26	-30.96	-30.82	-30.89	-29.59	-29.47	-29.53	-28.08	-27.92	-28.00
384	1.72	-40.08	-39.92	-40.00	-30.99	-30.75	-30.87	-29.67	-29.55	-29.61	-28.31	-28.21	-28.26
432	1.74	-40.21	-40.09	-40.15	-31.07	-30.87	-30.97	-29.84	-29.70	-29.77	-27.96	-27.86	-27.91
480	1.77	-40.06	-39.96	-40.01	-30.81	-30.65	-30.73	-29.72	-29.66	-29.69	-28.32	-28.28	-28.30
528	1.79	-40.06	-40.02	-40.04	-30.80	-30.78	-30.79	-29.47	-29.43	-29.45	-29.10	-29.04	-29.07
576	1.81	-40.21	-40.15	-40.18	30.99	-30.97	-30.98	-29.73	-29.57	-29.65	-29.30	-20.28	-29.29
624	1.83	-40.16	-40.04	-40.10	-30.58	-30.36	-30.47	-29.47	-29.39	-29.43	29.05	-28.81	-28.93
672	1.85	-40.18	-40.16	-40.17	-31.05	-30.97	-31.01	-29.63	-29.35	-29.49	29.41	29.17	-29.29
Kerogen Saergan plus water (water/ TOC=4)													
0	1.16	-41.82	-41.72	-41.77	-33.75	-33.51	-33.63	-30.81	-30.77	-30.79	-28.67	-28.51	-28.59
0	1.16	-41.85	-41.73	-41.79	-33.60	-33.54	-33.57	-30.96	-30.82	-30.89	--29.12	-28.96	-29.04
24	1.28	-41.74	-41.64	-41.69	-32.63	-32.45	-32.54	-30.72	-30.66	-30.69	-29.05	-28.77	-28.91
48	1.35	-41.59	-41.35	-41.47	-31.99	-31.98	-31.99	-30.72	-30.52	-30.62	-28.82	-28.66	-28.74
48	1.35	-41.70	-41.56	-41.68	-31.94	-31.86	-31.90	-30.42	-30.38	-30.40	-28.53	-28.47	-28.50
72	1.40	-41.60	-41.44	-41.52	-32.06	-31.86	-31.96	-30.54	-30.48	-30.51	-28.52	-28.34	-28.43
72	1.40	-41.56	-41.42	-41.49	-32.00	-31.84	-31.92	-30.63	-30.51	-30.57	-28.40	-28.22	-28.31
96	1.45	-41.69	-41.45	-41.57	-32.09	-32.07	-32.08	-30.82	-30.81	-30.81	-28.96	-28.62	-28.69
96	1.45	-41.98	-41.74	-41.86	-32.27	-32.11	-32.19	-30.45	-30.38	-30.41	-28.69	-28.41	-28.55

144	1.52	-40.93	-40.65	-40.79	-31.66	-31.54	-31.60	-30.37	-30.36	-30.37	-28.99	-28.75	-28.87
192	1.57	-40.06	-40.02	-40.04	-31.48	-31.34	-31.41	-30.77	-30.53	-30.65	-28.88	-28.64	-28.76
240	1.62	-40.38	-40.12	-40.25	-31.34	-31.35	-31.34	-30.19	-31.05	-30.12	-28.59	-28.49	-28.54
288	1.65	-40.32	-40.08	-40.20	-31.72	-31.56	-31.64	-30.34	-30.22	-30.28	-28.71	-28.63	-28.67
336	1.69	-39.81	-39.65	-39.73	-31.35	-31.29	-31.32	-29.99	-29.95	-29.97	-29.06	-29.78	-28.92
384	1.72	-38.83	-38.81	-38.82	-30.48	-30.36	-30.42	-29.23	-28.99	-29.11	-28.57	-28.67	-28.62
432	1.74	-39.15	-38.87	-39.01	-30.82	-30.78	-30.80	-29.48	-29.36	-29.42	-28.75	-28.51	-28.63
480	1.77	-39.55	-39.27	-39.41	-30.57	-30.51	-30.54	-29.08	-29.00	-29.04	-28.60	-28.52	-28.56
528	1.79	-39.51	-39.33	-39.42	-30.95	-30.79	-30.87	-29.41	-29.37	-29.39	-29.04	-28.98	-29.01
576	1.81	-39.79	-39.78	-39.79	-30.84	-30.82	-30.83	-29.81	-29.80	-29.81	-29.43	-29.35	-29.39
624	1.83	-39.18	-39.17	-39.18	-30.92	-30.80	-30.86	-29.43	-29.35	-29.39	-29.61	-29.49	-29.55
672	1.85	-39.12	-38.96	-39.04	-30.79	-30.73	-30.76	-29.54	-29.44	-29.49	-29.64	-29.48	-29.56
Kerogen Wuerhe													
0	1.21	-45.14	-45.29	-45.22	-34.16	-33.90	-34.03	-32.88	-32.60	-32.74	-28.52	-28.44	-28.48
0	1.21	-45.58	-45.48	-45.53	-34.00	-33.92	-33.96	-32.65	-32.61	-32.63	-28.46	-28.36	-28.41
48	1.37	-44.92	-44.88	-44.90	-33.40	-33.28	-33.34	-32.62	-32.65	-32.64	-26.86	-26.72	-26.79
96	1.47	-45.17	-44.93	-45.05	-33.29	-33.11	-33.20	-32.59	-32.35	-32.47	-26.92	-26.75	-26.84
96	1.47	-44.90	-44.74	-44.82	-33.32	-33.37	-33.35	-32.74	-32.42	-32.58	-27.11	-26.84	-26.98
144	1.53	-44.80	-44.93	-44.87	-33.10	-33.17	-33.13	-32.41	-32.54	-32.49	-26.71	-26.61	-26.66
192	1.58	-44.62	-44.38	-44.50	-33.58	-33.46	-33.52	-32.63	-32.46	-32.54	--26.82	-26.65	-26.73
192	1.58	-44.70	-44.67	-44.68	-33.92	-33.76	-33.84	-32.78	-32.76	-32.77	-26.87	-26.68	-26.78
240	1.63	-44.66	-44.42	-44.54	-34.10	-33.84	-33.97	-32.78	-32.70	-32.74	-27.04	-27.01	-27.02
288	1.66	44.17	-44.14	-44.15	-33.87	-33.85	-33.86	-32.74	-32.70	-32.72	-27.08	-26.98	-27.03
288	1.66	-45.01	-44.73	-44.87	-34.04	-34.00	-34.02	-32.92	-32.76	-32.84	-27.52	-27.34	-27.43
336	1.69	-44.44	-44.25	-44.35	-33.82	-33.72	-33.77	-32.73	-32.54	-32.63	-26.99	-29.91	-26.99
384	1.72	-43.47	-43.43	-43.45	-33.95	-33.93	-33.94	-32.77	-32.60	-32.69	-27.48	-27.34	-27.41

384	1.72	-44.27	-44.16	-44.22	-34.36	-34.14	-34.25	-33.06	-32.83	-32.95	-27.39	-27.13	-27.26
480	1.77	-44.09	-43.85	-43.97	-34.29	-34.21	-34.25	-32.97	-32.89	-32.92	-27.28	-27.14	-27.21
576	1.82	-43.60	-43.56	-43.58	-34.62	-34.61	-34.61	-33.12	-32.98	-33.05	-27.36	-27.30	-27.33
576	1.82	-43.36	-43.26	-43.31	-34.51	-34.39	-34.45	-33.02	-32.95	-32.99	-27.45	-27.39	-27.42
672	1.85	-43.42	-43.22	-43.32	-33.99	-34.09	-34.04	-32.79	-32.64	-32.72	-27.18	-27.19	-27.18
Kerogen Wuerhe plus water (water/ TOC=2)													
0	1.21	-45.55	-45.38	-45.47	-34.76	-34.65	-34.70	-33.84	-33.64	-33.74	-28.92	-28.75	-28.83
0	1.21	-45.75	-45.61	-45.68	-34.96	-34.89	-34.92	-34.04	-34.01	-34.02	-29.13	-29.12	-29.12
48	1.37	-45.13	-45.07	-45.10	-33.96	-33.82	-33.89	-33.27	-33.20	-33.23	-27.35	-27.18	-27.26
96	1.47	-45.27	-44.99	-45.13	-33.73	-33.47	-33.60	-33.22	-33.02	-33.12	-27.17	-26.99	-27.08
96	1.47	-44.91	-44.65	-44.78	-33.93	-3388	-33.90	-33.24	-33.23	-33.23	-27.18	-27.04	-27.11
144	1.53	-44.99	-44.73	-44.86	-33.99	-33.77	-33.88	-33.24	-33.08	-33.08	-27.56	-27.38	-27.47
192	1.58	-44.82	-44.58	-44.70	-33.83	-33.69	-33.76	-32.89	-32.71	-32.80	-27.18	-26.94	-27.06
192	1.58	-45.51	-45.23	-45.37	-34.13	-33.94	-34.03	-33.06	-32.84	-32.95	-27.18	-26.93	-27.06
240	1.63	-44.48	-44.28	-44.38	-34.06	-34.02	-34.04	-33.03	-32.86	-32.95	-27.58	-27.34	-27.46
288	1.66	-44.42	-44.16	-44.29	-33.68	-33.61	-33.65	-32.72	-32.70	-32.71	-27.02	-26.86	-26.94
288	1.66	-43.51	-43.41	-43.46	-33.54	-33.62	-33.58	-32.76	-32.55	-32.65	-26.94	-26.86	-26.90
336	1.69	-42.82	-42.68	-42.84	-33.96	-33.88	-33.92	-32.94	-32.82	-32.88	-27.32	-27.18	-27.25
384	1.72	-43.32	-43.24	-43.28	-34.16	-34.12	-34.14	-32.95	-32.81	-32.88	-27.58	-27.32	-27.45
384	1.72	-43.21	-42.98	-43.09	-33.99	-33.95	-33.97	-33.04	-32.87	-32.95	-27.50	-27.68	-27.59
480	1.77	-42.96	-42.74	-42.85	-34.05	-33.79	-33.92	-33.02	-32.84	-32.93	-27.49	-27.27	-27.38
576	1.82	-43.64	-43.40	-43.52	-34.80	-34.72	-34.76	-32.81	-32.73	-32.77	-27.82	-27.58	-27.70
576	1.82	-42.55	-42.37	-42.46	-34.26	-34.06	-34.16	-33.00	-32.72	-32.86	-27.31	-27.27	-27.29
672	1.85	-43.01	-42.81	-42.91	-34.64	-34.56	-34.60	-32.83	-32.75	-32.79	-28.02	-27.92	27.97
Kerogen Fengcheng													
0	1.07	-44.50	-44.23	-44.36	-32.62	-32.44	-32.53	-32.02	-31.87	-31.94	-23.71	-23.54	-23.63

48	1.32	-43.74	-43.50	-43.62	-31.27	-31.11	-31.19	-31.03	-30.83	-30.93	-23.49	-23.31	-23.40
48	1.32	-43.38	-43.12	-43.25	-30.89	-30.65	-30.77	-30.90	-30.74	-30.82	-23.50	-23.22	-23.36
96	1.43	-41.79	-41.64	-41.71	-30.08	-29.95	-30.01	-30.42	-30.28	-30.35	-22.84	-22.62	-22.73
144	1.50	-42.24	-41.99	-42.11	-30.66	-30.49	-30.57	-30.87	-30.67	-30.77	-23.07	-22.93	-23.00
144	1.50	-42.79	-42.69	-42.74	-30.68	-30.44	-30.56	30.91	-30.74	-30.82	-23.15	-23.00	-23.08
240	1.61	-41.55	-41.83	-41.69	-31.19	-30.93	-31.06	31.08	-31.04	-31.06	-22.71	-22.57	-22.64
240	1.61	-41.60	-41.52	-41.56	-31.05	-30.81	-30.93	-31.17	-31.05	-31.11	-22.74	-22.50	-22.62
288	1.65	-42.12	-41.82	-41.97	-30.70	-30.57	-30.64	-31.06	-30.96	-31.01	-22.75	-22.49	-22.62
336	1.68	-41.58	-41.28	-41.43	-30.82	-30.66	-30.74	-30.99	-30.98	-30.98	-22.54	-22.30	-22.42
336	1.68	-41.21	-41.25	-41.23	-30.48	-30.51	-30.50	-31.05	-30.91	-30.98	-22.75	-22.45	-22.60
384	1.71	-41.30	41.02	-41.16	-31.21	-31.07	-31.14	-30.92	-30.76	-30.84	-23.45	-23.19	-23.32
480	1.76	-40.88	-40.60	-40.74	--31.18	-31.08	-31.13	-31.09	-31.03	-31.06	-23.48	-23.22	-23.35
480	1.76	-40.33	-40.32	-40.33	-31.13	-31.11	-31.12	-30.85	-30.77	-30.81	-23.45	-23.41	-23.43
576	1.81	-40.96	-41.15	-41.06	-31.33	-31.32	-31.32	-31.08	-31.05	-31.06	-23.16	-23.08	-23.12
672	1.85	-40.84	-40.60	-40.72	-31.36	-31.26	-31.31	-30.88	-30.84	-30.86	-23.59	-23.33	-23.46
672	1.85	-40.50	-40.22	-40.36	-31.49	-31.38	-31.43	-30.48	-30.32	-30.40	-23.71	-23.46	-23.59
Kerogen Fengcheng plus water (water/ TOC=2)													
0	1.07	-44.92	-44.69	-44.81	-33.13	-33.07	-33.10	-32.64	-32.60	-32.62	-23.47	23.47	-23.47
48	1.32	-44.44	-44.14	-44.29	-31.74	-31.82	-31.78	-31.71	-31.68	-31.70	-22.70	-22.62	-22.65
48	1.32	-44.43	-44.16	-44.30	-31.90	-31.74	-31.81	-31.72	-32.05	-31.90	-22.68	-22.64	-22.66
96	1.43	-43.98	-43.70	-43.84	-31.58	-31.46	-31.52	-31.34	-31.20	-31.27	-22.86	-22.60	-22.73
144	1.50	-42.96	-42.74	-42.85	-31.63	-31.50	-31.56	-31.53	-31.45	-31.49	-22.85	-22.75	-22.80
144	1.50	-41.76	-41.50	-41.63	-31.59	-31.33	-31.46	-31.45	-31.25	-31.35	-23.27	-23.08	-23.17
192	1.56	-41.73	-41.75	-41.74	-31.48	-31.50	-31.49	-31.12	-30.97	-31.04	-23.07	-22.97	-23.02
240	1.61	-41.49	-41.29	-41.39	-31.24	-31.24	-31.24	-30.86	-31.00	-30.93	-23.34	-23.07	-23.20
240	1.61	-40.90	-40.66	-40.78	-30.69	-30.50	-30.59	-30.80	30.76	-30.78	-22.74	-22.52	-22.63

288	1.65	-41.14	-41.02	-41.08	-30.93	-30.71	-30.82	-31.05	-30.81	-31.34	-22.28	-22.20	-22.24
336	1.68	-40.89	-40.65	-40.77	-31.21	-31.03	-31.12	-30.73	-30.49	-30.61	-23.46	-23.23	-23.35
336	1.68	-40.64	-40.49	-40.56	-31.50	31.22	-31.36	-30.79	30.73	-30.36	-23.39	-23.35	-23.37
384	1.71	-39.83	-39.69	-39.76	-31.34	-31.14	-31.24	-30.65	-30.62	-30.63	-23.93	-23.87	-23.90
480	1.76	-39.63	-39.37	-39.50	-31.50	-31.37	-31.43	-30.44	-30.46	-30.45	-24.22	-24.06	-24.14
480	1.76	-39.78	-39.58	-39.68	-31.45	-31.39	-31.42	-30.59	-30.50	-30.54	-24.06	-23.98	-24.02
576	1.81	-39.25	-39.17	-39.21	-31.83	-31.60	-31.71	-30.50	-30.45	-30.48	-24.27	-24.23	-24.25
672	1.85	-39.36	-39.19	-39.28	-31.60	-31.42	-30.51	-30.12	-30.24	-30.18	-24.40	-24.19	-24.29
672	1.85	-39.95	-39.96	-39.95	-31.79	-31.75	-31.77	-30.78	-30.52	-30.65	-24.41	-24.30	-24.35

1, 2 and 3: data obtained from the first, second and third measurements; av: averaged value
